# Comparative analysis of tuberous root metabolites between cultivated and wild varieties of *Rehmannia glutinosa* by widely targeted metabolomics

**DOI:** 10.1038/s41598-021-90961-6

**Published:** 2021-06-01

**Authors:** Yanqing Zhou, Luying Shao, Jialin Zhu, Huimin Li, Hongying Duan

**Affiliations:** College of Life Sciences, Henan Normal University, Xinxiang, 453007 Henan People’s Republic of China

**Keywords:** Genetics, Plant sciences

## Abstract

Differential metabolites between tuberous roots from cultivated variety (ZP) and wild variety (YS) of *Rehmannia glutinosa* were analyzed by widely targeted metabolomics, and annotated to KEGG pathways. 228 secondary metabolites (SM) in ZP and YS were detected, of which 58 were differential metabolites (DM), including 41 flavonoids, 10 phenolic acids, 3 terpenoids, 2 alkaloids and 2 others, and 170 were unchanged; Among 58 DMs, 44 (75.9%) were up-regulated in YS, of which 30 were unique to YS, while 14 (24.1%) were down-regulated in YS, of which 10 were unique to ZP; Among flavonoids, 33 (80.5%) were more highly expressed in YS than in ZP; Among phenolic acids, 7 (70%) were more highly expressed in YS than in ZP; 12 of 58 DMs were annotated into 17 types of KEGG pathways. Among them, benzoic acid and p-Coumaryl alcohol were up-regulated in YS, and annotated into 10 pathways (58.8%) and 4 pathways (23.5%), respectively. In addition, much of DMs possess various pharmacological effects. These results indicated better quality of YS than ZP and the necessity of YS domestication. Taken together, this study will provide a reference for the scientific introduction, comprehensive development and utilization of wild *Rehmannia glutinosa*.

## Introduction

*Rehmannia glutinosa* Libosch. (*R. glutinosa*) is a perennial herb belonging to the genus *Rehmannia* (Scrophulariaceae), and has important economic value as medicinal and food materials. Its tuberous root is a commonly used bulk Chinese herbal medicine. The pharmaceutical effects of *R. glutinosa* are closely related to its secondary metabolites. Many metabolites in its tuberous root have been identified up to date. For example, 1049 metabolites were identified from the developing tuberous root of its variety Jinjiu^1^. They are composed of catalpol, acteoside, saccharides, terpene glycosides, amino acids, trace elements and other components, which possesses some bioactivities such as anti-cardiovascular diseases, nerve protection, hypertension resistance, immunity enhancement and so on^2–4^. *R. glutinosa* is mainly distributed in some provinces, such as Henan, Shanxi, Shandong and others, in China. Among them, better *Rehmanniae* Radix is from cultivated *R. glutinosa* in Huaiqing region including Wen County, Wuzhi County, Boai County and Jiaozuo city, Henan, which has higher contents of bioactive components such as catalpol and verbascoside, and lower clinical dosage than that from other places^5^. Its commonly cultivated varieties are Beijing No.3, Wen 85-5, Jinjiu, etc. In recent years, with its increasing consumption and loss of its farmland, its resources become increasingly exhausted. Moreover, its variety complexity and long-term vegetative propagation make its varieties degenerated, which lead to its poor quality, low yield and narrow genetic basis^6^. Therefore, it urgently requires new germplasms. To our knowledge, one practical solution to its new germplasms is the introduction, comprehensive development and utilization of wild *R. glutinosa*.

Wild *R. glutinosa* resources with many good traits and genes are very important and significant for enriching *R. glutinosa* germplasm resources and improving the yield and quality of cultivated *R. glutinosa* varieties, but their qualities vary. As a result, they need be identified and evaluated before their development and utilization. SNP-based *R. glutinosa* germplasms analysis indicated that there were more significant differences of its cultivated varieties from its wild resources^7^. Moreover, compared with wild *Rehmannia* germplasms, the qualities of cultivated *R. glutinosa* varieties decrease in that their some genes were lost via long-term artificial selection. Accurate chemical composition analysis of *R. glutinosa* varieties is crucial to their quality evaluation. Therefore, the study of chemical composition of wild and cultivated *R. glutinosa* will help to solve the lack of its new germplasms. Metabolomics, an ongoing and practical technology, has been widely used in medicine development, medicine toxicity and mechanism research, medicine screening and efficacy evaluation, mining new secondary metabolites, plant metabolism and response mechanisms, microbial interactions, gene function elucidation, effective metabolic pathways and related regulatory mechanisms^1,8–14^. At present, metabolomic technologies include targeted metabolomics, untargeted metabolomics and widely targeted metabolomics. So far, the first two metabolomic technologies have widely been used in plants, including *R. glutinosa*^15–20^, but both have advantages and disadvantages. Widely targeted metabolomics is next generation metabolomics, combining the advantages of untargeted metabolomics and targeted metabolomics, and possesses some advantages such as qualitative and quantitative accuracy, high throughput, high sensitivity and wide coverage. Using Q-TRAP mass spectrometry based on MRM mode, hundreds of known metabolites and nearly a thousand unknown metabolites can be simultaneously quantified, and the detection and identification of highly sensitive and widely targeted metabolites come true^21,22^. In recent years, this technology has been successfully applied in *Sesame*^23^, *Rice*^22,24,25^, *Vanilla*^26^ and *Chrysanthemum morifolium*^27^, respectively.

In our study, widely targeted metabolomics was used to compare the metabolic profiles between ZP and YS of *R. glutinosa* for the first time in order to qualitatively and quantitatively identify differential metabolites between ZP and YS. The results of this study will provide a reference for the scientific introduction, comprehensive development and utilization of wild *R. glutinosa***.**

## Results

### Phenotypic analysis

There were some significant differences between ZP and YS in phenotype before the propagation of wild *R. glutinosa* (Table 1). However, the metabolite profiles of these two varieties were unknown and needed further analysis in this study.

**Table 1:** Phenotypic analysis of two varieties.

Variety	Wild type	Beijing No.3
Elevation	184 m	97.10 m
Root number per plant	2–4	4.00
Fresh weight of the heaviest root per plant (g)	2.10	100
Growing period (year)	Natural growth for many years	Vegetative reproduction for many years
Growing environment	Natural mountain soil	Cultivated soil
Tuberous root color	Dark yellow	Bright yellow

### Multivariate analysis

Multivariate analysis is the analysis of three or more variables. It is used to deal with the relationship between variables, including different methods such as Principal components analysis (PCA). Orthogonal Partial Least Squares-Discriminant Analysis (OPLS-DA) and so on. PCA and OPLS-DA were used in this study.

### PCA

**Figure 1:**
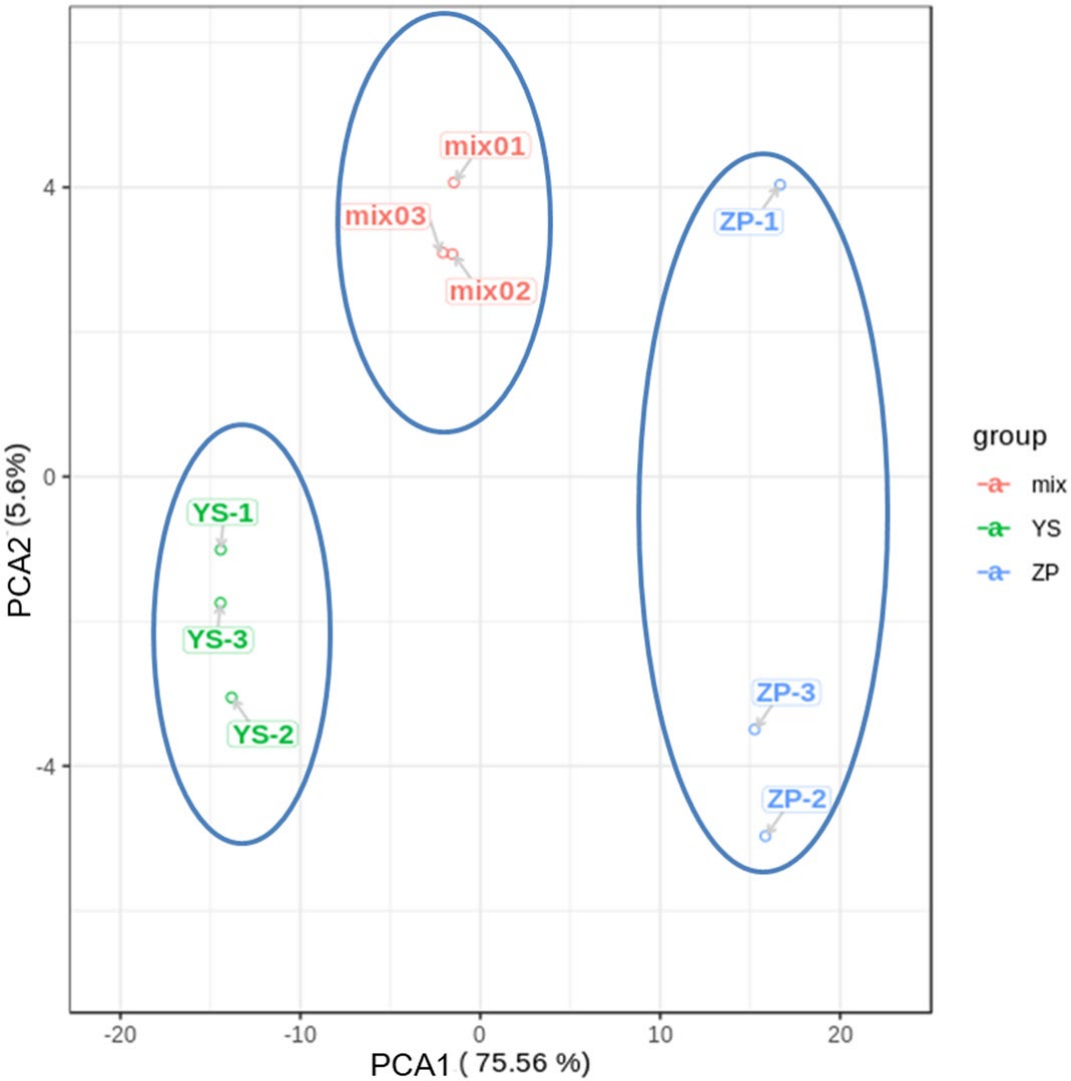
PCA score chart based on mass spectrum data of ZP, YS and QC samples. Ordinate: the second principal component, Abscissa: the first principal component. PCA Plot visualized in ggplot2 toolkit of R v3.5.0 (https://www.r-project.org/).

PCA was used to reveal the internal structure of several variables by a few principal components. In the PCA plot (Fig. 1), three mix samples as QC were grouped together, suggesting QC samples with similar metabolic profiles and the entire analysis with stability and repeatability. Meanwhile, it could be seen from Fig. 1 that YS samples were clustered together, located to the left side of the QC samples, and that ZP samples gathered together, located on the right side to the QC samples. These results indicated that our analysis was stable and repeatable.

### OPLS-DA

**Figure 2:**
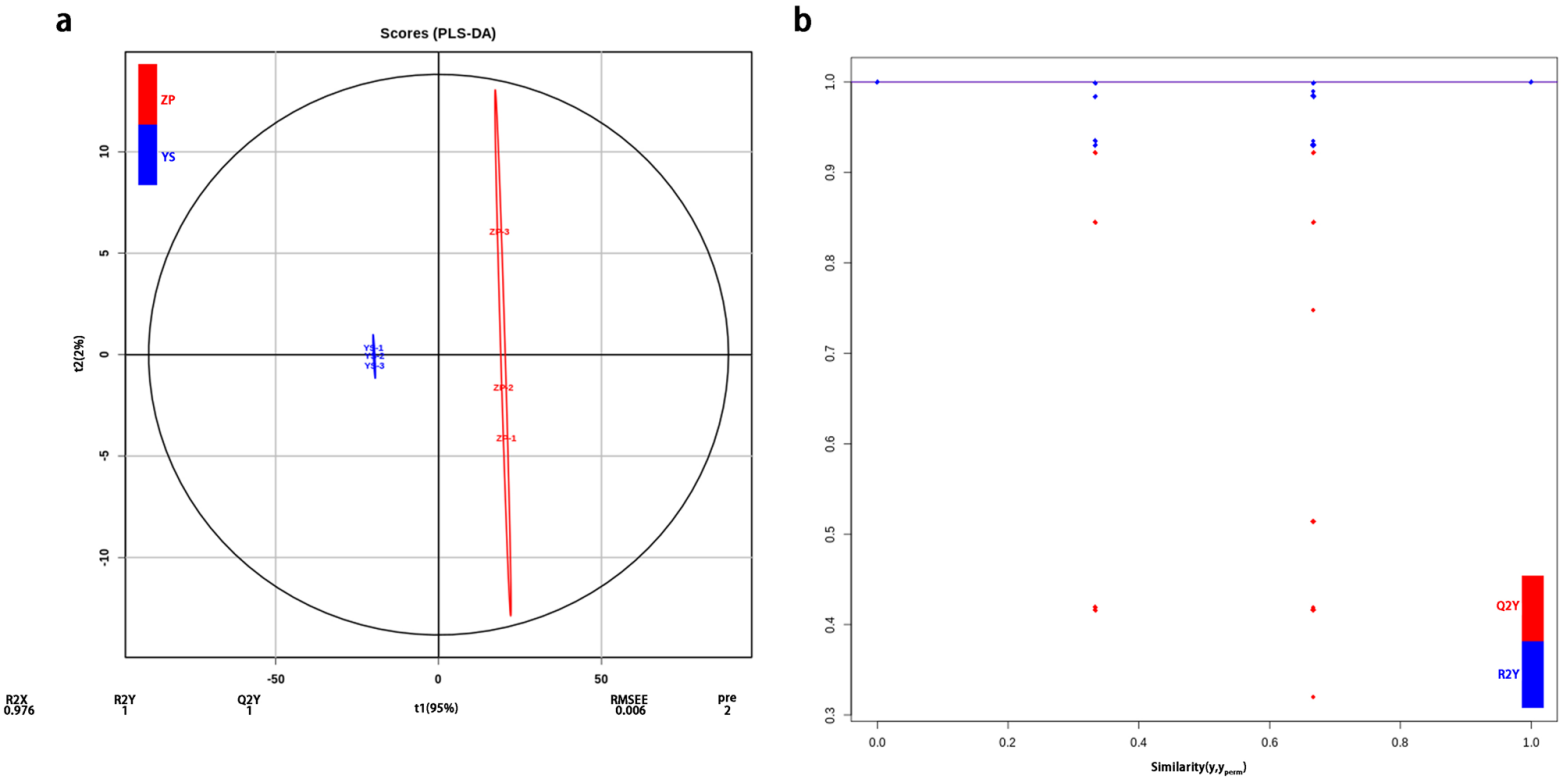
(**a**) OPLS-DA score Plot. t1: Predicted principal components-score value of main components and difference between observation groups, t01: Orthogonal principal component-score value of orthogonal components and difference in observation group, R^2^Y: Percentage of Y matrix information that can be explained by OPLS-DA, Q^2^Y (Q^2^): Prediction ability of OPLS-DA, Pre: Predicted principal component number, RMSEE: Root mean squared error, Red: ZP, Blue: YS. (**b**) OPLS-DA permutation verification plot. Red: Q^2^Y, Blue: R^2^Y. OPLS-DA score Plot visualized in ggplot2 toolkit of R v1.0.1 (https://www.r-project.org/).

OPLS-DA is a multivariate statistical analysis method with supervised pattern recognition, and can solve the problem that PCA is not sensitive to the variables with little correlation. According to the differential variables, the score plot of every component (Fig. 2a) was formed to further show the differences between the components^28^. It was seen from Fig. 2a that R^2^Y and Q^2^ Y(Q^2^) were 1, while R^2^X equals 0.976, suggesting that OPLS-DA should be stable and reliable. Because Q^2^ equals 1, more than 0.9, OPLS-DA is excellent. The OPLS-DA model is verified using 200 alignment experiments. The horizontal line corresponds to the R^2^Y and Q^2^ of the original model, while the red and blue dots represent R^2^Y and Q^2^ after replacement, respectively. R^2^Y (0.81) and Q^2^ (0.52) in Fig. 2b were smaller than R^2^Y (1) and Q^2^ (1) of the original model, suggesting that the corresponding points should not exceed the corresponding lines. Therefore, OPLS-DA is meaningful, from which variable infuence in projection (VIP) values are obtained. VIP value is used to screen differential metabolites.

### Identification of differential metabolites

A total of 228 SMs between YS and ZP were detected by HMDB, MWDB and METLIN databases (see Supplementary Table S1 online). Using both FC ≥ 2 or ≤ 0.5 and VIP ≥ 1 as the screening standards, 58 DMs were  screened and  identified  (Table 2).

**Table 2:** DMs from 228 SMs.

No	Formula	Compounds	VIP	Fold change	p-value	Type
1	C_29_H_34_O_16_	Limocitrin-O-rhamnoside-O-rhamnoside	1.329	102.477	0.002	Up
2	C_31_H_48_O_7_	Phytolaccagenin	1.929	0.000	0.000	Down
3	C_14_H_19_NO_7_	Ehretioside	1.895	12,259.259	0.000	Up
4	C_16_H_12_O_6_	Aracarpene 1	1.952	21,777.778	0.004	Up
5	C_27_H_28_O_17_	Kaempferol 3-glucuronide-7-glucoside	1.857	8529.630	0.008	Up
6	C_27_H_26_O_17_	Apigenin-7-O-diglucuronide	2.074	79,666.667	0.005	Up
7	C_17_H_14_O_7_	Tricin	1.681	1644.444	0.001	Up
8	C_27_H_30_O_15_	Kaempferol glc-rha	1.926	16,814.815	0.007	Up
9	C_16_H_12_O_6_	6,7,8-Tetrahydroxy-5-methoxyflavone	1.852	8066.667	0.004	Up
10	C_21_H_18_O_11_	Baicalin	1.166	34.653	0.004	Up
11	C_16_H_12_O_6_	Diosmetin	1.950	21,444.444	0.002	Up
12	C_27_H_31_ClO_15_	Pelargonin chloride	1.804	0.000	0.007	Down
13	C_9_H_10_O_2_	p-Coumaryl alcohol	1.028	15.936	0.000	Up
14	C_20_H_39_NO_2_	N-Oleoylethanolamine	1.035	16.587	0.000	Up
15	C_33_H_40_O_19_	Robinin(kaempferol-3-O-gal-rham-7-O-rham)	1.832	0.000	0.170	Down
16	C_31_H_38_O_16_	2′-Acetylacteoside	1.195	0.023	0.029	Down
17	C_16_H_22_O_10_	Geniposidic acid	1.329	0.010	0.001	Down
18	C_28_H_32_O_15_	Diosmin	1.234	47.068	0.000	Up
19	C_9_H_12_O_3_	Homovanillic alcohol	1.683	1718.519	0.017	Up
20	C_22_H_23_O_11_ +	Peonidin O-hexoside	1.804	5107.407	0.006	Up
21	C_23_H_24_O_13_	Syringetin 3-O-hexoside	1.125	0.036	0.002	Down
22	C_28_H_32_O_16_	Chrysoeriol O-hexosyl-O-hexoside	1.836	6937.037	0.004	Up
23	C_26_H_26_O_15_	Tricin O-malonylhexoside	1.896	12,407.407	0.000	Up
24	C_23_H_24_O_12_	Tricin 7-O-hexoside	1.164	33.997	0.001	Up
25	C_17_H_22_O_10_	1-O-β-d-Glucopyranosyl sinapate	1.910	0.000	0.012	Down
26	C_28_H_32_O_15_	Chrysoeriol 7-O-rutinoside	1.902	13,111.111	0.002	Up
27	C_22_H_20_O_12_	Chrysoeriol O-glucuronic acid	1.440	228.916	0.005	Up
28	C_23_H_22_O_13_	Tricin O-glucuronic acid	2.081	85,814.815	0.006	Up
29	C_22_H_28_O_13_	3-O-p-coumaroyl quinic acid O-hexoside	1.604	859.259	0.006	Up
30	C_22_H_26_O_12_	5-O-p-Coumaroyl shikimic acid O-hexoside	1.749	3048.148	0.005	Up
31	C_15_H_10_O_6_	Luteolin	1.521	452.222	0.037	Up
32	C_7_H_6_O_2_	Benzoic acid	1.937	18,703.704	0.002	Up
33	C_21_H_20_O_10_	Kaempferol 7-O-rhamnoside	1.708	0.000	0.196	Down
34	C_7_H_6_O_3_	Protocatechuic aldehyde	1.918	15,407.407	0.003	Up
35	C_27_H_30_O_14_	Kaempferol 3,7-dirhamnoside (kaempferitrin)	2.059	0.000	0.151	Down
36	C_22_H_23_O_11_ +	Peonidin 3-O-glucoside	1.797	4762.963	0.003	Up
37	C_27_H_30_O_16_	Bioquercetin	1.571	0.001	0.046	Down
38	C_27_H_30_O_14_	Kaempferol-3,7-O-α-l-rhamnoside	1.872	0.000	0.108	Down
39	C_23_H_32_O_15_	β-d-Furanofructosyl-α-d-(3-mustard cyl)glucoside	1.029	15.965	0.000	UP
40	C_16_H_24_O_7_	3-Hydroxy-4-isopropylbenzylalcohol 3-glucoside	1.986	31,074.074	0.001	Up
41	C_21_H_18_O_11_	Apigenin-7-O-β-d-glucuronide	1.220	49.046	0.006	Up
42	C_21_H_18_O_12_	Tetahydroxy-flavone-7-O-β-d-glucuronide	2.006	38,074.074	0.000	Up
43	C_16_H_12_O_6_	Hispidulin	1.854	8237.037	0.003	Up
44	C_17_H_14_O_7_	Jaceosidin	1.632	1081.111	0.002	Up
45	C_21_H_18_O_12_	Scutellarin	2.008	39,037.037	0.001	Up
46	C_24_H_22_O_13_	Malonyglygenistin	1.505	40.364	0.000	Up
47	C_16_H_12_O_6_	Pratensein	1.856	8407.407	0.004	Up
48	C_25_H_18_O_9_	Luteolin-7-O-glucuronide	1.608	60.303	0.003	Up
49	C_22_H_22_O_11_	Diosmetin-7-O-galactoside	1.016	14.960	0.001	Up
50	C_22_H_20_O_12_	Diosmetin-7-O-glucuronide	1.388	156.084	0.004	Up
51	C_25_H_24_O_14_	Diosmetin-7-O-(6′-O-malonyl)-β-d-glucoside	1.672	1533.333	0.003	Up
52	C_27_H_30_O_15_	Luteolin-7-O-rutinoside	1.873	0.000	0.165	Down
53	C_22_H_30_O_14_	3′-O-d-glucosylgentiopicroside	1.694	1862.963	0.003	Up
54	C_15_H_22_O_8_	Bartsioside	1.977	28,333.333	0.002	Up
55	C_30_H_38_O_15_	Cistanoside C	1.044	0.056	0.038	Down
56	C_22_H_34_NO_10_ +	Sinapine glucoside	1.828	0.000	0.315	Down
57	C_28_H_28_O_18_	Chrysoeriol-7-O-[β-d-glucuronopyranosyl- (1 → 2)-O-β-d-glucuronopyranoside]	2.067	74,185.185	0.013	Up
58	C_27_H_26_O_17_	Apigenin-7-O-[β-d-glucuronopyranosyl(1 → 2)-O-β-d-glucuronopyranoside)	2.109	116,407.407	0.002	Up

Up: compared with ZP, the corresponding metabolite was up-regulated in YS. Down: compared with ZP, the corresponding metabolite was down-regulated in YS.

### Display of the difference data between groups by Volcano plot

**Figure 3:**
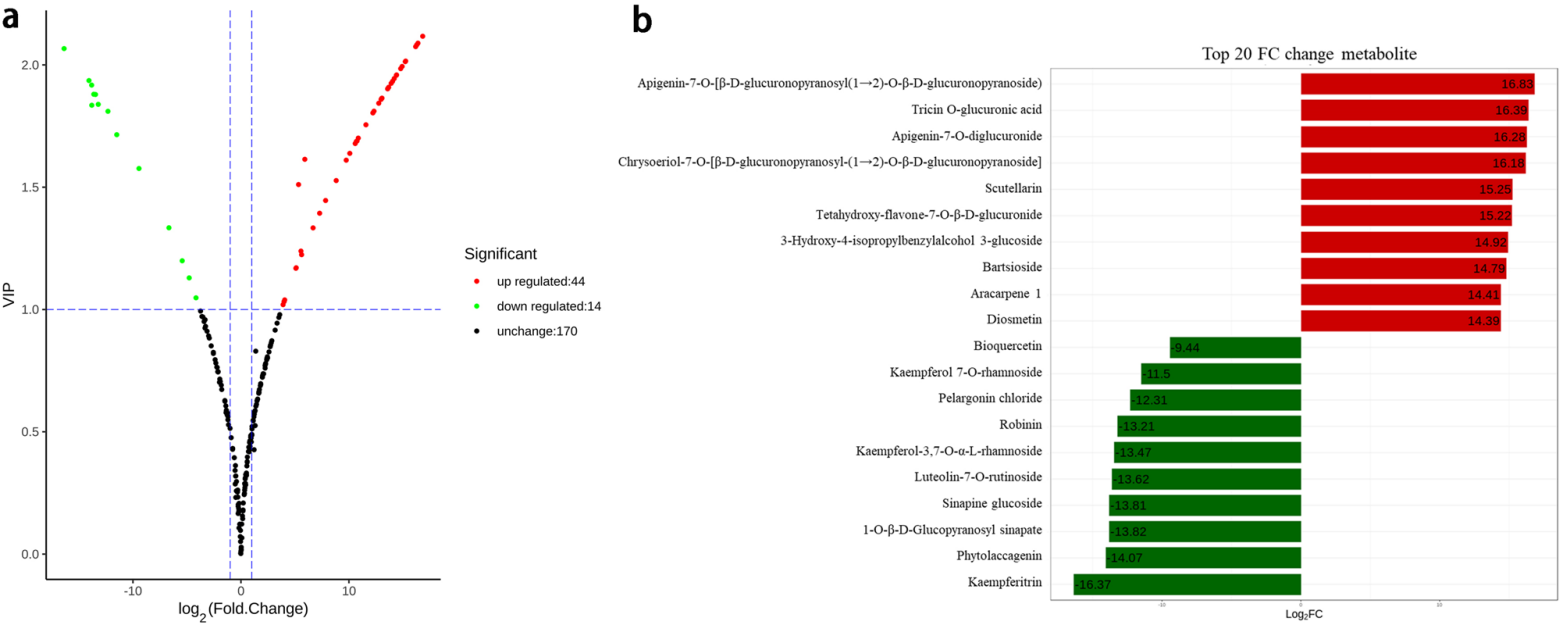
(**a**) Volcano Plot of differential metabolites. Abscissa: FC value, Ordinate: VIP value, The dots: differential metabolites, Green dots: down regulated metabolites, Red dots: up regulated metabolites, Black dots: detected metabolites without significant difference. Volcano Plot visualized in ggplot2 toolkit of R v3.5.0 (https://www.r-project.org/). (**b**) Top 20 FC change metabolites. Ordinate: metabolite, Blackish green color: down regulated metabolites, Red: up regulated metabolites.

Based on log_2_ FC value and p-value, DMs were displayed in Volcano plot (Fig. 3a). In Fig. 3a, there were 44 up-regulated metabolites indicating that their expression contents in YS were higher than that in ZP, 14 down-regulated metabolites indicating that their expression contents in ZP were higher than that in YS, and 170 unchanged metabolites indicating that their expression contents did not vary between YS and ZP. Meanwhile, Top 20 FC change metabolites were presented in Fig. 3b. This result was consistent with that based on the VIP values and FCs (Table 2).

### Heatmap clustering

**Figure 4:**
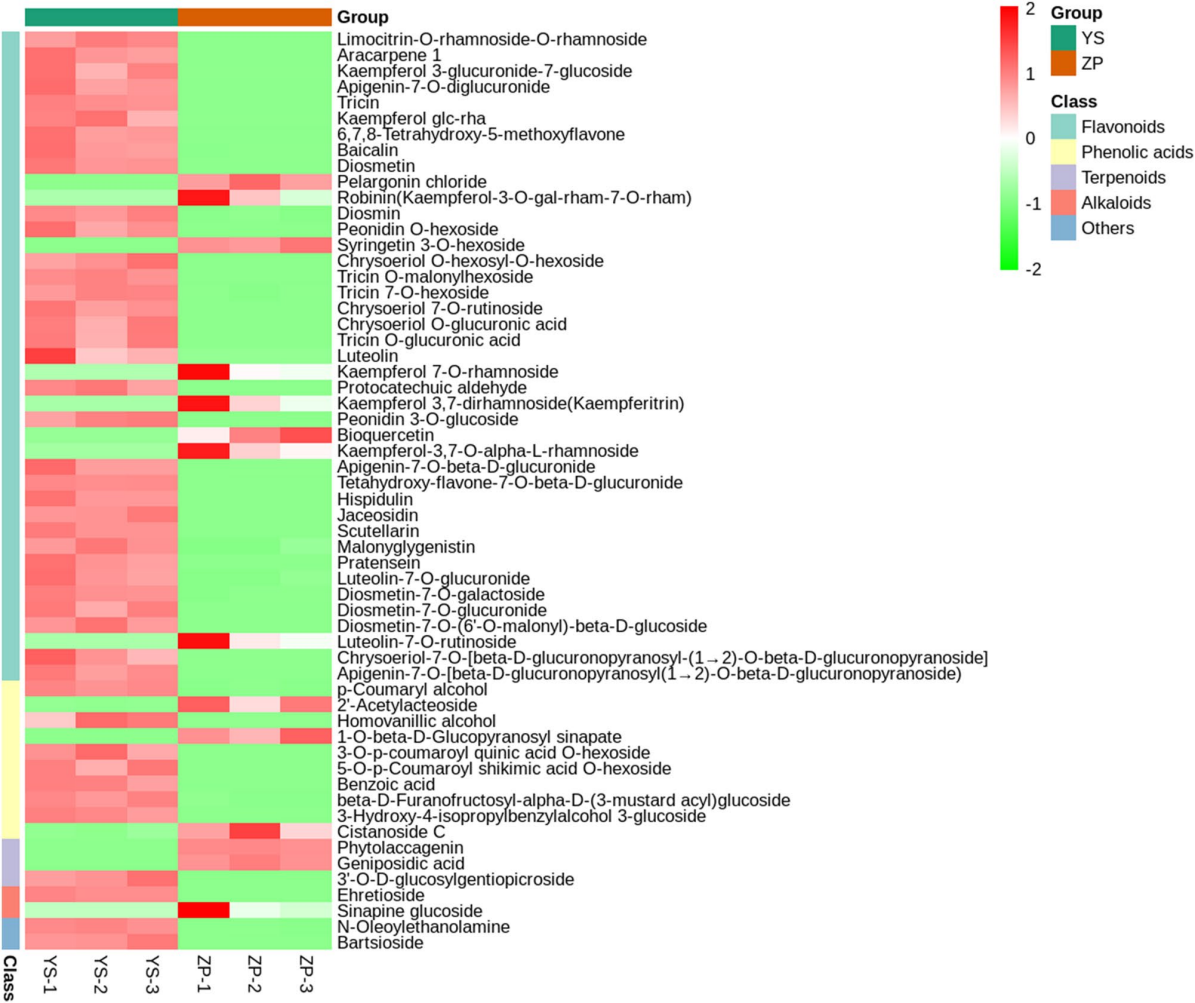
Heat map of 58 DMs. Ordinate: clustering after normalization of relative contents of different metabolites, 5 kinds of colors stand for 5 classes of metabolites, Abscissa: grouping of samples, 2 kinds of colors stand for 2 groups of samples, Color scale from green to red indicated the contents of differentially expressed metabolites vary from low to high. Heatmap visualized in ggplot2 toolkit of R v1.0.12 (https://www.r-project.org/).

In order to show the varying law of 58 DMs with significant differences, their heat map was drawn (Fig. 4). The results showed that three YS repeats were grouped into one category and three ZP repeats were grouped into the other category. Because different metabolites had different accumulation trends in different samples, and the closer the accumulation trends, the closer the distances, 58 DMs had obvious expression differences between ZP and YS. 58 DMs were grouped into 5 clusters: (1) flavonoids including 41 ones, which were dominant in the SM of *R. glutinosa* tuberous roots. Among them, 33 (80.5%) metabolites were expressed higher in YS than in ZP. (2) 10 phenolic acids, of which 7 (70%) metabolites were expressed higher in YS than in ZP. (3) 3 terpenoids, 1 (33.33%) of which was expressed higher in YS than in ZP. (4) 2 alkaloids, half of which was higher expressed in YS than in ZP. (5) 2 metabolites that were higher expressed in YS than in ZP. In total, 44 differential metabolites (75.9%) were higher expressed in YS than in ZP, which was consistent with the screening of DMs based on VIP and FC as well as by Volcano plot (Table 2, Fig. 3b).

### Pharmaceutical activities of DMs suggesting quality changes

It was seen from Fig. 4 that DMs were grouped into 5 types such as flavonoids, phenolic acids, terpenoids, alkaloids and and others. Among them, flavonoids including luteolin, protocatechuic aldehyde and tricin, were a large family of plant secondary metabolites and a subdivision of polyphenols, a versatile class of natural compounds that represented secondary metabolites from higher plants. Flavonoids were an effective ingredient of many Chinese herbal medicines and had various medicinal effect, including antibacterial, anti-inflammatory, anti-oxidant^29,30^. Phenolic acids including benzoic acid and p-Coumaryl alcohol, had been proved to have a variety of pharmacological activities, such as cardiovascular and cerebrovascular effects, anti-tumor, anti-oxidation, anti-inflammation, anti-fibrosis, etc^31^. Terpenoids including phytolaccagenin, geniposidic acid, were also a kind of important compounds in Chinese herbal medicine and played an important role in plant growth and development, resistance and defense, etc^32^. Alkaloids including ehretioside were a kind of nitrogen-containing basic organic compounds existing in nature (mainly plants). Alkaloids had many pharmacological activities, such as analgesia, spasmolysis, anti-inflammation, anti-tumor, etc. In addition, some compounds in alkaloids could interact with chemical components such as saponins and terpenoids, which could achieve the pharmacological effects of relieving cough, eliminating phlegm and relieving asthma^33^.

### Differential metabolic pathways

**Figure 5:**
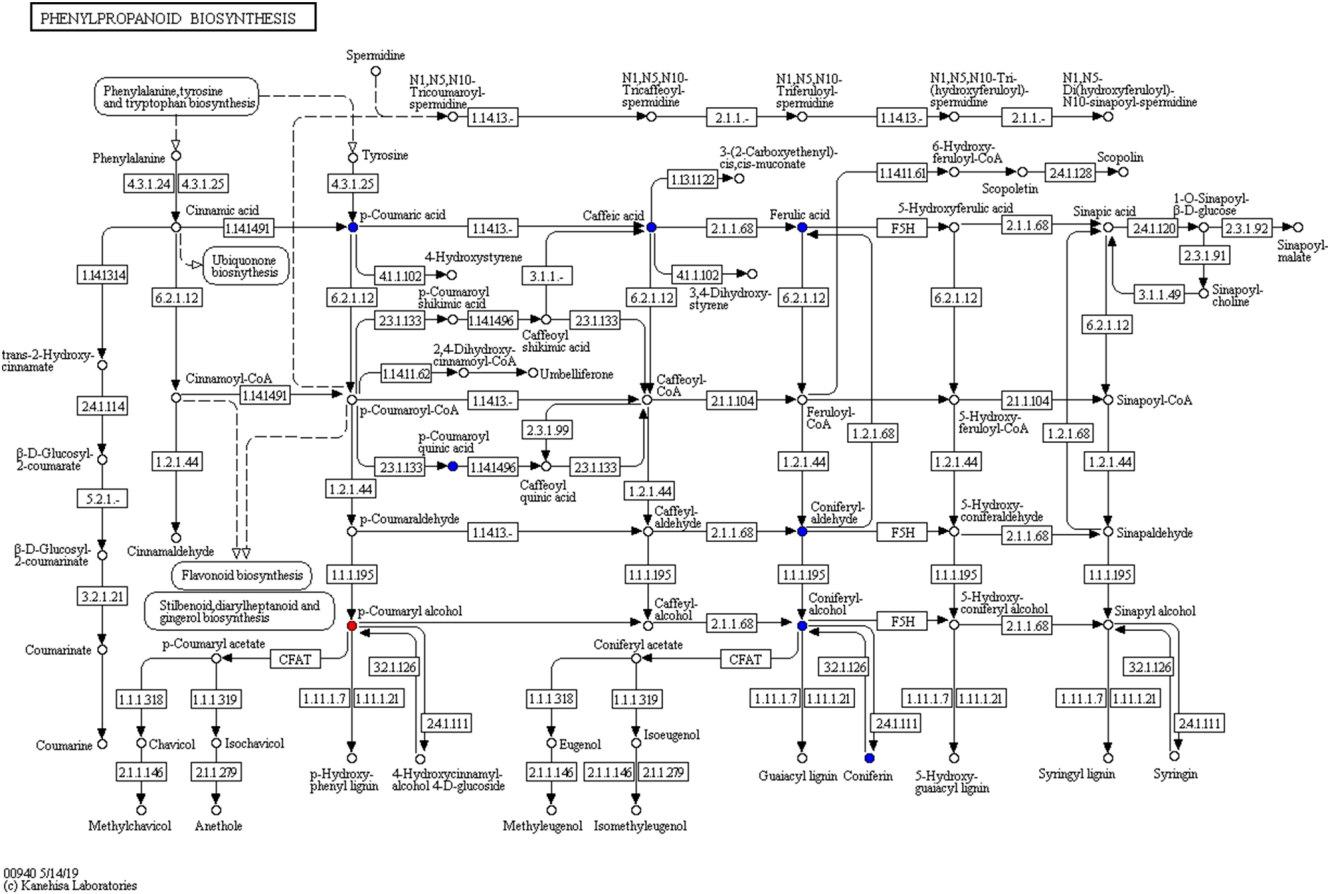
Phenylpropanoid biosynthesis. The red dots: the differentially expressed metabolites that are increased, the blue dots: the detected metabolites, but there is no significant difference.

When 228 SMs were annotated to KEGG pathways by KEGG database (https://www.kegg.jp/kegg/kegg1.html)^34^, 38 SMs were annotated to KEGG pathways 202 times. Their annotation times ranged from 1 times per metabolite to 33 times per metabolite (succinic acid). After some duplicate KEGG pathways were removed, there were still 77 KEGG pathways (see Supplementary Table S2 online). Take Phenylpropanoid biosynthesis of KEGG pathway as an example (Fig. 5). In Fig. 5, p-Coumaric acid, p-coumaryl alcohol, caffeic acid, ferulic acid, coniferyl aldehyde, coniferyl alcohol, p-coumaroyl quinic acid and coniferin were annotated in Phenylpropanoid biosynthesis, of which p-coumaryl alcohol was a differential metabolite up-regulated in YS, compared with ZP, but p-Coumaric acid, caffeic acid, ferulic acid, coniferyl aldehyde, coniferyl alcohol, p-coumaroyl quinic acid and coniferin were unchanged.

Among 58 DMs, 12 were annotated to KEGG database, 7 of which were annotated to KEGG pathways 23 times (see Supplementary Table S3 online). After some duplicate KEGG pathways were removed, there were still 17 KEGG pathways (Fig. 5). The seven metabolites were pelargonin chloride, peonidin 3-O-glucoside, p-Coumaryl alcohol, luteolin, benzoic acid, protocatechuic aldehyde and pratensein, respectively. These 17 KEGG pathways were categories anthocyanin biosynthesis, phenylpropanoid biosynthesis, biosynthesis of phenylpropanoids, metabolic pathways, biosynthesis of secondary metabolites, flavonoid biosynthesis, flavone and flavonol biosynthesis, phenylalanine metabolism, benzoate degradation, dioxin degradation, toluene degradation, aminobenzoate degradation, biosynthesis of alkaloids derived from shikimate pathway, microbial metabolism in diverse environments, degradation of aromatic compounds, isoquinoline alkaloid biosynthesis and isoflavonoid biosynthesis. Among them, phenylpropanoid biosynthesis and biosynthesis of phenylpropanoids were different metabolic pathway names in KEGG, which had similar names, but corresponded to two different pathway IDs (Table 3).

**Table 3:** The categories of 12 DMs-annotated KEGG pathways.

KEGG pathway	Ko-ID	Number	DM	Cpd-ID
Anthocyanin biosynthesis	ko00942	2	Pelargonin chloride; peonidin 3-O-glucoside	C08725 + C12141
Phenylpropanoid biosynthesis	ko00940	1	p-Coumaryl alcohol	C02646
Biosynthesis of phenylpropanoids	ko01061	1	p-Coumaryl alcohol	C02646
Metabolic pathways	ko01100	3	Luteolin; p-coumaryl alcohol; benzoic acid	C01514 + C02646 + C00180
Biosynthesis of secondary metabolites	ko01110	3	Benzoic acid; luteolin; p-coumaryl alcohol	C00180 + C01514 + C02646
Flavonoid biosynthesis	ko00941	1	Luteolin	C01514
Flavone and flavonol biosynthesis	ko00944	1	Luteolin	C01514
Phenylalanine metabolism	ko00360	1	Benzoic acid	C00180
Benzoate degradation	ko00362	1	Benzoic acid	C00180
Dioxin degradation	ko00621	1	Benzoic acid	C00180
Toluene degradation	ko00623	1	Benzoic acid	C00180
Aminobenzoate degradation	ko00627	1	Benzoic acid	C00180
Biosynthesis of alkaloids derived from shikimate pathway	ko01063	2	Benzoic acid; protocatechuic aldehyde	C00180 + C16700
Microbial metabolism in diverse environments	ko01120	1	Benzoic acid	C00180
Degradation of aromatic compounds	ko01220	1	Benzoic acid	C00180
Isoquinoline alkaloid biosynthesis	ko00950	1	Protocatechuic aldehyde	C16700
Isoflavonoid biosynthesis	ko00943	1	Pratensein	C10520

Ko-ID = ID of KEGG pathway, Number = the number of metabolites that can be annotated to the corresponding KEGG pathways, Cpd-ID = Number of compound in KEGG.

## Discussion

*Rehmannia glutinosa* is an important perennial herb, and its tuberous roots is clinically used to treat fever, nervous conditions, diabetes, and hypertension, and to increase liver function, hematopoietic function and immune defense and so on in different ways^1^. The great medicinal market demand for *R. glutinosa* leads to its overexploitation and germplasm resource scarcity, and long-term vegetative propagation makes the variety degenerate, quality poor, yield low and genetic basis narrow, so it is urgent to enrich and improve *R. glutinosa* by using candidate germplasm resources. Wild *R. glutinosa* is a kind of important candidate resources for this objective. Before we make good use of its wild resources, we used widely targeted metabolomics to compare the SM between its cultivated and wild varieties in that its SM are vital for its important pharmacological activities.

On the one hand, we compared the phenotypic characteristics of ZP and YS. It was found that both differences were very obvious, especially in both sizes and weights (Table 1). This was consistent with that of Zuo et al.^35^. On the other hand, 228 SMs were detected in both YS and ZP using widely targeted metabolomics technology coupled with MRM and public databases (see Supplementary Table S1 online). They were divided into 170 unchanged metabolites and 58 DMs (44 upregulated, 14 downregulated) (Table 2, Fig. 3b). 58 DMs were divided into 5 types such as flavonoids (41), phenolic acids (10), terpenoids (3), alkaloids (2) and others (2) (Table 2).

It is known that plant metabolomes are composed of over 200,000 metabolites that control plant development, and even *Arabidopsis* contains some 5000 metabolites^1^, so *R. glutinosa* should contain more than these 228 SMs in YS and ZP of *R. glutinosa* Libosch. Moreover, 1049 metabolites were ever identified in the developing tuberous roots of *R. glutinosa* variety Jinjiu^36^. Therefore, the high throughout identification of widely targeted metabolomics used in this study was limited, compared with untargeted metabolomics^36^ and others^37,38^. Its limitation should be attributed to the lack of large herbal medicine public metabolite databases.

The SMs of medicinal plants are very important substances in their life activities, closely related to their defense against diseases and insect pests and environmental stress, and important pharmacological activities. Based on these, our DMs may reflect the differences between wild and cultivated varieties of *R. glutinosa*. In this study, (1) the number of upregulated metabolites (44) were bigger than that of downregulated metabolites (14) in YS, compared to ZP (Table 2, Fig. 3b), Among 41 flavonoids, 33 (80.5%) were more highly expressed in YS than ZP, while among 10 phenolic acids, 7 (70%) were more highly expressed in YS than ZP. The number of unique metabolites to YS (30) was bigger than that of unique metabolites to ZP (10). Because these DMs possess important effects, these results suggested that the quality of YS is better than that of ZP. For example, flavonol had antioxidation; luteolin had a variety of pharmacological effects such as anti-tumor, antibacterial, anti-inflammatory, antiviral and analgesic effects^39^. Anthocyanin, a water-soluble flavonoid compound, had a variety of biological functions such as protecting plants from UV damage, scavenging reactive oxygen species, resisting adversity and changing the color of plants, as well as health efficacy such as anti-aging, anti-obesity and prevention of cardiovascular diseases^40^. According to investigation and research, flavonoids were synthesized by using intermediate products from phenylalanine converted via phenylpropane route as synthetic precursors, which were formed by different synthetic routes. Due to their various pharmacological effects such as cardiovascular protection, anti-cancer, anti-oxidation, anti-inflammation, liver protection and anti-tumor etc. flavonoids had become hot spots in the development and research of natural medicine at home and abroad^27,41,42^. According to our results, the expression of flavonoids and phenolic acids in YS was much higher; (2) Among quality control standards of *R. glutinosa* such as catalpol and rehmannioside D (now) or verbascoside (ever) in the Pharmacopoeia of the People’s Republic of China^43^, rehmannioside D, catalpol and verbascoside were all contained in unchanged. These result showed that YS could be used to modify ZP, of which catalpol′ result is consistent with a previous report^44^; (3) In this study, as the autotoxic metabolites such as ferulic acid, benzoic acid, protocatechuic aldehyde, 4-Hydroxybenzoic acid and so on, their the contents in YS were also much higher than that in ZP. Among them, benzoic acid had antibacterial and antiseptic effects^45^, ferulic acid could inhibit obesity and improve the steady state of blood lipid and blood sugar^46,47^, and protocatechuic aldehyde had cardiovascular and cerebrovascular protective effects^48^. Moreover, 30 kinds of DMs such as protocatechuic aldehyde, luteolin, tricin, diosmetin, homovanillic alcohol, jaceosidin, pratensein, hispidulin, malonyglygenistin, bartsioside, scutellarin and other compounds were unique to YS and reported for the first time in *R. glutinosa*. These results indicate ZP and YS contained unique DMs, unique ones to YS were much more than that to ZP, and that artificial breeding increased the contents of main active metabolites in cultivated variety of *R. glutinosa* and selected out many metabolite in its wild variety.

**Figure 6:**
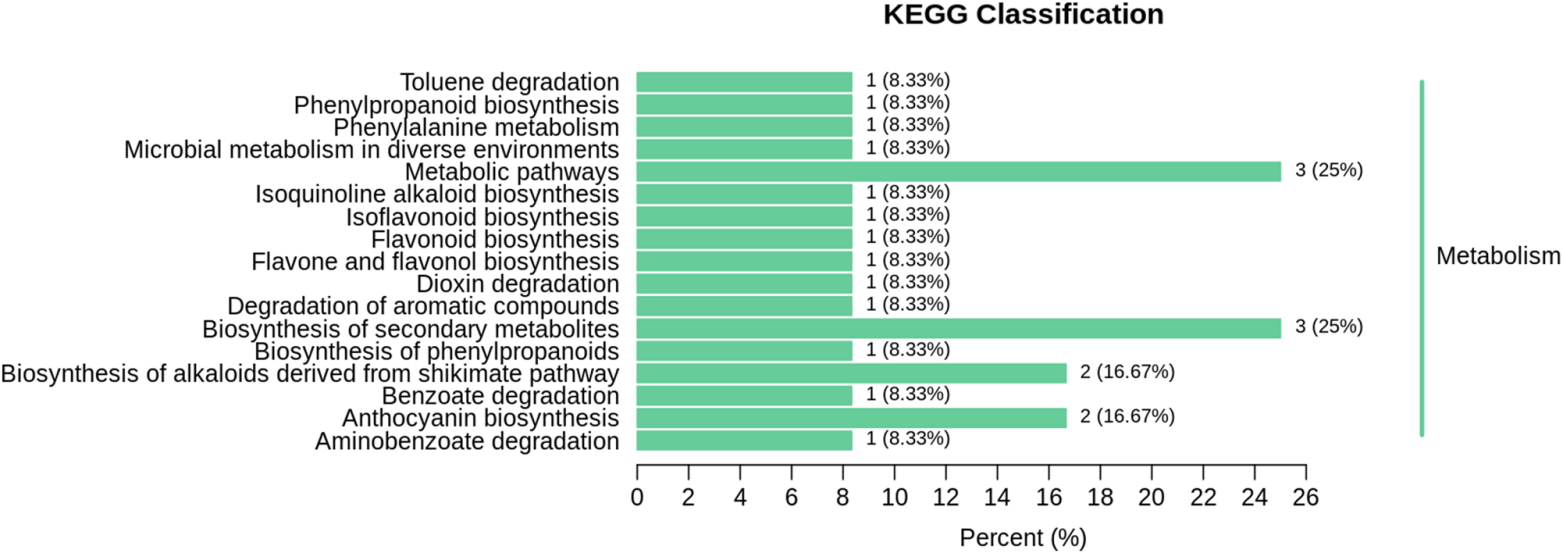
KEGG pathway classification map of DMs. Ordinate: KEGG metabolic pathway types. Abscissa: the number of DMs annotated to the KEGG pathway type and the proportion of this number to the total number of all the DMs annotated to all the KEGG pathways.

In addition, KEGG database helps researchers to study genes, expression information and metabolite content as a whole network, and provides integrated metabolic pathways involved in such as the pathways of carbohydrate, nucleoside and amino acids as well as biodegradation of organic compounds with enzymes. Therefore, it is a powerful tool for metabolism analysis and metabolic network research in vivo^1,49^. In the present study, 12 of 58 DMs between ZP and YS were annotated to 17 non-repetitive KEGG pathways (Fig. 6, Table 3). The main differential metabolic pathways between ZP and YS included metabolic pathways and biosynthesis of secondary metabolites. Among them, benzoic acid and p-Coumaryl alcohol up-regulated in YS were annotated into 10 (58.8%) and 4 (23.5%), respectively (Table 3). These results provided a clue for analyzing the metabolism of these metabolites and DMs, and their metabolic networks in *R. glutinosa.* In conclusion, based on the phenotypic differences between YS and ZP, we detected 228 SMs of YS and ZP by using widely targeted metabolomics, and then identified 58 DMs between ZP and YS via multivariate analysis. It was found that the metabolites of YS were more unique, and some of them were quality control metabolites in YS instead of ZP. Our results indicated better quality of YS than that of ZP and the necessity of YS domestication, and will provide a reference for the scientific introduction, comprehensive development and utilization of wild *R. glutinosa*. In addition, the related metabolic pathways will provide a theoretical basis for the subsequent exploration of biosynthesis of related metabolites in *R. glutinosa*.

## Materials and methods

### Plant materials

The plant materials of *R. glutinosa* were provided by Four huaiyao research institutes in Wuzhi county, Jiaozuo city, Henan province on November 10, 2019. Among materials, the cultivated variety (ZP) of *R. glutinosa* was collected from germplasm resource nursery of Four huaiyao research institutes and the wild variety (YS) of *R. glutinosa* was collected from Sumen mountain located in Wuzhi county, Jiaozuo city, Henan province. Relevant Permissions had been provided. Then, two varieties were planted in experimental field of Henan Normal University. All samples were collected with approval and permission. Both tuberous roots were collected and freeze–dried for metabolites profiling. Three samples were used, of which each was composed of 6 different tuberous roots from 6 different mature plants in their harvest time. In each sample, they were pooled in equal parts after collection. These samples were metabolically profiled at Wuhan Maitville Biotechnology Co., Ltd (http://www.metware.cn).

### Statement about the wild plant collected

Collection of *Rehmannia glutinosa* in this research material conforms to and complies with the IUCN Policy Statement on Research Involving Species at Risk of Extinction and the Convention on the Trade in Endangered Species of Wild Fauna and Flora. In addition, according to the List of National Key Protected Wild Plants issued by the State Forestry and Grassland Bureau of China, *Rehmannia glutinosa*, the experimental material of this study, is neither a national key protected wild plant nor an endangered plant species.

### Sample preparation and extraction

Related experiments such as sample preparation and extraction of metabolites were conducted by Wuhan Maitville Biotechnology Co., Ltd. Gradient grades of methanol, acetonitrile, and acetic acid were purchased from Merck Company, Germany (http://www.merck-chemicals.com). The internal standard lidocaine was bought from Shanghai New Asiatic Pharmaceuticals Co., Ltd (http://www.xinyapharm.com/). Authentic standards of phenylpropanoids were purchased from BioBioPha Co., Ltd (http://www.biobiopha.com/) and all other standards were from Sigma-Aldrich, USA (http://www.sigmaaldrich.com/united-states.html). The vacuum freeze- dried samples (three biological replicates) were ground into powder using a mixer mill (MM 400, Retsch) with a zirconia bead for 1.5 min at 30 Hz. 100 mg powder was weighed and extracted overnight at 4 °C with 1.0 ml pure methanol (or 70% aqueous methanol) containing 0.1 mg/L lidocaine for lipid-solubility metabolites (or water-solubility metabolites). After centrifuged at 10,000*g* for 10 min, the supernatants were absorbed, and filtrated by millipore filter (SCAA-104, 0.22 μm pore size; ANPEL, Shanghai, China, http://www.anpel.com.cn/) and stored in vials for UPLC-MS/MS analysis^38^.

### Widely targeted metabolomics conditions

Data acquisition instrument system: Ultra Performance Liquid Chromatography (UPLC) (Shim-pack UFLC SHIMADZU CBM30A, http://www.shimadzu.com.cn/) and Tandem mass spectrometry, MS/MS (Applied Biosystems 4500 QTRAP, http://www.appliedbiosystems.com.cn/). Liquid chromatographic conditions: Column- Waters ACQUITY UPLC HSS T3 C18 (1.8 µm, 2.1 mm * 100 mm); solvent system, water (0.04% acetic acid): acetonitrile (0.04% acetic acid); gradient program, 100:0 V/V at 0 min, 5:95 V/V at 11.0 min, 5:95 V/V at 12.0 min, 95:5 V/V at 12.1 min, 95:5 V/V at 15.0 min; flow rate, 0.40 mL/min^50^; temperature, 40 °C; injection volume: 5 μL. The effluent was alternatively connected to an ESI-triple quadrupole-linear ion trap (Q TRAP)-MS.

LIT and triple quadrupole (QQQ) scans were acquired on a triple quadrupole-linear ion trap mass spectrometer (Q TRAP), API 4500 Q TRAP LC/MS/MS System, equipped with an ESI Turbo Ion-Spray interface, operating in a positive ion mode and controlled by Analyst 1.6.3 software (AB Sciex). The ESI source operation parameters were as follows: ion source, turbo spray; source temperature 550 °C; ion spray voltage (IS) 5500 V; ion source gas I (GSI), gas II(GSII), curtain gas (CUR) were set at 55, 60, and 25.0 psi, respectively; the collision gas(CAD) was high. Instrument tuning and mass calibration were performed with 10 and 100 μmol/L polypropylene glycol solutions in QQQ and LIT modes, respectively. QQQ scans were acquired as MRM experiments with collision gas (nitrogen) set to 5 psi. DP and CE for individual MRM transitions were done with further DP and CE optimization. A specific set of MRM transitions were monitored for each period according to the metabolites eluted within this period. Similar but inconsistent experimental procedures had been successfully applied and implemented by Zhang et al.^51^ before.

### Qualitative and quantitative analyses of metabolites

Metabolite structure analysis referred to existing mass spectrum public databases such as MassBank (http://www.massbank.jp/), KNAPSAcK (http://kanaya.naist.jp/KNApSAcK/), HMDB (http://www.hmdb.ca/)^52^, MoTo DB (http://www.ab.wur.nl/moto/) and METLIN (http://metlin.scripps.edu/index.php)^53^ and others. The primary and secondary spectra detected by mass spectrometry were analyzed qualitatively, and isotopic signals were removed during the analysis of some substances, including repeated signals of K^+^ ions, Na^+^ ions, NH^4+^ ions, and fragment ions that were themselves other larger molecular weight substances repeating signal; Metabolites were quantified using MRM mode for mass spectrum peaks of metabolites, a peak per metabolite (mass spectrum file). Mass spectrum file was processed by MultiaQuant software for integration and correction of chromatographic peaks, a chromatographic peak per metabolite^54^.

### Statistical data analysis

Metabolite data were log2-transformed for statistical analysis to improve normality and normalized. In order to explore the metabolites of cultivated and wild *R. glutinosa*, the 228 SMs had been used for cluster analysis by R v3.5.0 (http://www.r-project.org/). The cluster heat map were obtained using the agglomeration method of ‘complete linkage’ based on the Euclidean distances of 228 SMs between accessions. The color scale indicates the intensity of the metabolites (log2-transformed). Peak areas were integrated using the IntelliQuan algorithm^50^. Differences in the metabolites of root tissue between ZP and YS were determined using Welch’s t-test (P < 0.01). The significantly changed (P < 0.01) metabolites were used for subsequent PCA. In parallel, unsupervised PCA was carried out by R v3.5.0 (https://www.r-project.org/). The supervised OPLS-DA was carried out by R v1.0.1, MetaboAnalystR (https://www.r-project.org/)^51^. Data were processed using Analyst 1.6.3 software.

### KEGG function annotation

DMs were annotated to KEGG pathways by KEGG database^34^. These KEGG pathways were classified according to their types.

### Compliance with ethical standards

The conducted experiment complies with the laws of China.

## Supplementary Information

Supplementary Information.
