# Supplementary material for: Comparative analysis of tuberous root metabolites between cultivated and wild varieties of Rehmannia glutinosa by widely targeted metabolomics
Source: Sci Rep. 2021 Jun 1;11:11460. doi: 10.1038/s41598-021-90961-6 (PMC8169854; doi:10.1038/s41598-021-90961-6)
Supplement: Supplementary file 1 — Supplementary Information. [file 41598_2021_90961_MOESM1_ESM.pdf]

Comparative Analysis of Tuberous Root Metabolites between Cultivated and Wild Varieties of *Rehmannia Glutinosa* by Widely Targeted Metabolomics

Yanqing Zhou [1]*, Luying Shao[1 #], Jialin Zhu[1 #], Huimin Li[1], Hongying Duan[1]

[1]College of Life Sciences, Henan Normal University, Xinxiang, 453007, HN, P.R.China
***corresponding. Author @ yqzhou@htu.cn**
[#] these authors contributed equally to this work

Table. S1. Information of 228 secondary metabolites

No.	Q1 (Da)	Molecular Weight (Da)	Ionization model	Formula	Compounds	Class	mix01	mix02	mix03	YS-1	YS-2	YS-3	ZP-1	ZP-2	ZP-3
1	623.1	624.14	[M-H]-	C28H32O16	Isorhamnetin-3-O-rutinoside	Flavonoids	1680000	1900000	1710000	407000	406000	421000	3130000	2970000	2220000
2	331.07	332.075	[M-H]-	C13H16O10	3-O-Galloyl-β-D-glucose	Tannins	145000	183000	181000	136000	301000	302000	110000	99700	86900
3	331.07	332.075	[M-H]-	C13H16O10	2-O-Galloyl-β-D-glucose	Tannins	546000	536000	594000	145000	211000	206000	720000	893000	1030000
4	169.05	168.042	[M+H]+	C8H8O4	Gallacetophenone	Others	9320	10400	7490	12700	14000	11600	10700	5630	11900
5	507.17	508.179	[M-H]-	C21H32O14	6'-O-glucosylaucubin	Others	80300	123000	86800	12300	21900	17400	203000	206000	184000
6	487.15	488.152	[M-H]-	C21H28O13	Cistanoside F	Phenolic acids	1130000	1170000	1090000	609000	646000	594000	1980000	2580000	1970000
7	639.19	638.184	[M+H]+	C29H34O16	Limocitrin-O-rhamnoside-O-rhamnoside	Flavonoids	282000	313000	321000	545000	632000	602000	5900	6050	5410
8	227.13	226.114	[M+H]+	C14H14N2O	Crenatine	Alkaloids	167000	154000	194000	220000	249000	253000	64700	83300	79100
9	461.17	462.174	[M-H]-	C20H30O12	Verbasoside	Phenolic acids	2080000	2220000	2170000	1700000	1970000	1900000	2190000	2680000	3240000
10	637.18	638.184	[M-H]-	C29H34O16	β-Oxoacteoside	Others	163000	167000	142000	25000	25000	31900	371000	396000	233000

11	651.23	652.237	[M-H]-	C31H40O15	Cistanoside D	Others	6240000	6360000	7170000	3440000	3270000	3220000	10500000	14600000	8950000
12	625.21	624.205	[M+H]+	C29H36O15	Isoacteoside	Others	1820000	1840000	1720000	230000	318000	416000	3320000	4350000	2020000
13	179.07	178.063	[M+H]+	C10H10O3	4-Hydroxy-3-methoxy cinnamaldehyde	Phenolic acids	339000	367000	374000	462000	474000	436000	205000	252000	275000
14	487.35	488.35	[M-H]-	C30H48O5	Madasiatic acid	Terpenoids	4320000	4330000	4650000	936000	733000	980000	8180000	8950000	11100000
15	471.35	472.355	[M-H]-	C30H48O4	Corosolic acid	Terpenoids	522000	496000	501000	129000	132000	145000	980000	1280000	1320000
16	387.2	386.194	[M+H]+	C19H30O8	Roseoside	Flavonoids	60000	48300	67100	63700	61100	72200	47300	80500	73100
17	531.33	532.338	[M-H]-	C31H48O7	Phytolaccagenin	Terpenoids	61300	66300	82800	9	9	9	155000	157000	152000
18	315.11	316.116	[M-H]-	C14H20O8	5-(2-Hydroxyethyl)-2-O-glucosylohenol	Phenolic acids	392000	375000	353000	358000	419000	386000	392000	413000	387000
19	168.07	167.059	[M+H]+	C8H9NO3	4,5,6-Trihydroxy-2-cyclohexen-1-ylideneacetonitrile	Alkaloids	384000	415000	419000	453000	430000	455000	426000	414000	431000
20	314.12	313.117	[M+H]+	C14H19NO7	Ehretioside	Alkaloids	90100	72200	84800	113000	109000	109000	9	9	9
21	297.12	298.12	[M-H]-	C18H18O4	Tababiphenyl C	Others	47200	62900	49600	35900	43400	46800	57600	46100	54100
22	427.06	426.028	[M+H]+	C17H14O13	Ditartaroyl-hydroxylcoumarin	Phenolic acids	584000	626000	646000	705000	684000	699000	733000	658000	708000
23	519.11	520.1	[M-H]-	C24H24O13	Isorhamnetin acetyl hexoside	Flavonoids	25900	19800	22100	23500	26300	25300	11700	9590	12900
24	451.1	452.076	[M-H]-	C20H20O12	Maleoyl-caffeoylquinic acid	Phenolic acids	53400	63500	55200	91100	93000	91000	11900	16300	13400
25	485.36	486.337	[M-H]-	C31H50O4	Ursolic acid-OCH3	Terpenoids	4540	4670	4930	2300	1910	2370	9790	8650	7090
26	309.06	310.069	[M-H]-	C14H14O8	Feruloylmalic acid	Phenolic acids	253000	276000	252000	70000	67600	58100	466000	512000	514000

27	299.06	300.063	[M-H]-	C16H12O6	Aracarpene 1	Flavonoids	149000	159000	153000	220000	187000	181000	9	9	9
28	675.41	679.419	[M-H]-	C38H60O10	3-O-(2-O-Acetyl-β-D-glucopyranosyl) oleanolic acid	Terpenoids	11900000	12800000	10000000	12900000	16100000	13100000	4440000	6050000	5020000
29	463.12	462.116	[M+H]+	C22H22O11	Methylquercetin rha	Flavonoids	12100	11200	16400	12700	14000	15100	26900	8340	7840
30	579.21	580.216	[M-H]-	C28H36O13	Syringaresinol-4′-O-β-D-monO-glucoside	Lignans and Coumarins	177000	213000	140000	189000	250000	156000	89600	114000	92300
31	623.13	624.133	[M-H]-	C27H28O17	Kaempferol 3-glucuronide-7-glucoside	Flavonoids	35800	37700	49400	86900	63600	79800	9	9	9
32	621.11	622.117	[M-H]-	C27H26O17	Apigenin-7-O-diglucuronide	Flavonoids	427000	392000	415000	817000	649000	685000	9	9	9
33	331.08	330.074	[M+H]+	C17H14O7	Tricin	Flavonoids	11200	11000	15100	15500	14600	14300	9	9	9
34	595.16	594.158	[M+H]+	C27H30O15	Kaempferol glc-rha	Flavonoids	73600	95800	100000	158000	170000	126000	9	9	9
35	591.21	592.186	[M-H]-	C29H36O13	(+)-Medioresinol-aceGlu	Lignans and Coumarins	198000	195000	213000	315000	305000	299000	70400	63700	57400
36	519.19	520.168	[M-H]-	C26H32O11	Pinoresinol-Hexose	Lignans and Coumarins	334000	381000	401000	198000	212000	203000	592000	609000	520000
37	561.2	562.177	[M-H]-	C28H34O12	Pinoresinol-acetylglucose	Lignans and Coumarins	277000	258000	279000	384000	325000	333000	121000	133000	118000
38	579.21	580.186	[M-H]-	C28H36O13	Syringaresinol-Hex	Lignans and Coumarins	350000	370000	382000	370000	346000	350000	405000	389000	410000
39	621.21	622.195	[M-H]-	C30H38O14	Syringaresinol-aceGlu	Lignans and	252000	232000	230000	283000	280000	238000	176000	157000	203000

						Coumarins								
40	345.17	346.163	[M-H]-	C16H26O8	Rehmapicroside	Others	1670000	1730000	1830000	2270000	2090000	2040000	1030000	1100000	1010000
41	496.34	497.19	[M-H]-	C23H31NO11	Rehmaglutoside D	Terpenoids	1370000	1400000	1360000	1100000	1090000	1080000	1670000	2230000	1650000
42	325.18	324.15	[M+H]+	C16H24O5N2	Anabasine-Hexose	Alkaloids	66700	70500	69700	52500	59800	64200	65700	58300	67000
43	282.28	281.249	[M+H]+	C18H35NO	Octadecenoic amide	Others	96700	93800	91900	275000	323000	195000	86700	104000	217000
44	325.09	326.1	[M-H]-	C15H18O8	p-Coumaric acid-O-glycoside	Phenolic acids	829000	880000	874000	1110000	1320000	1090000	354000	324000	337000
45	355.1	356.11	[M-H]-	C16H20O9	Feruloyl glucose	Phenolic acids	236000	249000	222000	222000	227000	250000	323000	320000	351000
46	385.11	386.121	[M-H]-	C17H22O10	Isosinapic acid-hexoside	Phenolic acids	288000	290000	313000	430000	438000	413000	67800	88900	78400
47	301.07	300.063	[M+H]+	C16H12O6	6,7,8-Tetrahydroxy-5-methoxyflavone	Flavonoids	56900	60000	58000	81700	67300	68800	9	9	9
48	179.06	178.055	[M+H]+	C10H10O3	Coniferaldehyde	Phenolic acids	32600	37000	24000	45300	41300	50100	20400	27600	19100
49	193.06	194.049	[M-H]-	C10H10O4	Ferulic acid	Phenolic acids	1040000	976000	1220000	1340000	1410000	1310000	505000	534000	625000
50	203	202.197	[M+H]+	C10H26N4	Spermine	Alkaloids	1260000	1630000	1410000	1690000	1650000	1760000	1620000	1640000	1370000
51	197.05	198.044	[M-H]-	C9H10O5	Syringic acid	Phenolic acids	272000	260000	311000	373000	386000	375000	93700	109000	117000
52	167	168.035	[M-H]-	C8H8O4	Vanillic acid	Phenolic acids	1720000	1380000	1570000	2130000	1800000	2200000	312000	373000	489000
53	447.09	446.068	[M+H]+	C21H18O11	Baicalin	Flavonoids	935000	1050000	1040000	1980000	1650000	1620000	28100	55900	67500
54	299.06	300.053	[M-H]-	C16H12O6	Diosmetin	Flavonoids	150000	155000	145000	209000	184000	186000	9	9	9
55	179.08	180.07	[M-H]-	C10H12O	Coniferyl alcohol	Phenolic	2910000	2610000	2570000	3690000	3530000	3280000	975000	1190000	1050000

				3		acids								
56	355.13	356.11	[M-H]-	C20H20O6	(+)-Piperitol	Lignans and Coumarins	27100	26400	27700	35400	36000	46100	3710	4110	5280
57	357.14	358.125	[M-H]-	C20H22O6	Pinoresinol	Lignans and Coumarins	473000	437000	453000	633000	635000	622000	96100	93100	94300
58	160.05	161.042	[M-H]-	C9H7NO2	Indole-5-carboxylic acid	Alkaloids	35800	33500	34200	42000	39600	42800	22800	26400	17500
59	146.1	145.047	[M+H]+	C9H7NO	Indole-3-carboxaldehyde	Alkaloids	69000	81900	77800	83000	89400	70000	41000	52200	76500
60	595.17	630.107	[M-Cl]+	C27H31ClO15	Pelargonin chloride	Flavonoids	14300	16500	13400	9	9	9	42200	53400	41800
61	153	154.021	[M-H]-	C7H6O4	2,5-Dihydroxybenzoic acid	Phenolic acids	150000	139000	126000	128000	122000	135000	121000	216000	218000
62	117.03	118.021	[M-H]-	C4H6O4	Succinic acid	Others	4760000	4510000	5310000	5290000	5960000	5790000	3070000	4100000	3340000
63	133	134.015	[M-H]-	C4H6O5	L-(-)-Malic acid	Others	1300000	1590000	1820000	1210000	1170000	1340000	1420000	1740000	2010000
64	191	192.018	[M-H]-	C6H8O7	Citric Acid	Others	12300000	11200000	11800000	11400000	8520000	8330000	8340000	9140000	9150000
65	165.05	166.038	[M-H]-	C5H10O6	D-Xylonic acid	Others	5360000	5130000	5270000	7750000	6960000	6930000	4510000	4260000	4300000
66	151.05	152.041	[M-H]-	C8H8O3	Vanillin	Phenolic acids	6530000	5820000	6160000	9280000	8260000	9050000	1180000	1340000	1450000
67	122.1	121.082	[M+H]+	C8H11N	Phenethylamine	Others	75700	73200	82400	72100	75900	65400	71400	148000	77400
68	121.04	122.032	[M-H]-	C7H6O2	4-Hydroxybenzaldehyde	Phenolic acids	165000	155000	140000	222000	216000	215000	73400	98800	77900
69	219	218.095	[M+H]+	C12H14N2O2	N-Acetyl-5-hydroxytryptamine	Alkaloids	95400	104000	87400	71100	68600	61200	154000	216000	145000
70	140	141.011	[M-H]-	C2H8NO4P	O-Phosphorylethanolamine	Others	23700	26100	26000	9410	8460	6700	48900	41200	38600
71	319.05	320.041	[M-H]-	C15H12O8	Dihydromyricetin(Ampelopsin)	Flavonoids	44700	38200	31700	21600	29000	30800	43700	31900	38800
72	137.03	138.026	[M-H]-	C7H6O3	4-Hydroxybenzoic acid	Phenolic	396000	387000	384000	430000	427000	456000	248000	301000	435000

						acids									
73	341	342.113	[M-H]-	C16H22O8	Coniferin	Phenolic acids	3440000	3120000	3520000	1270000	1290000	1120000	5370000	7410000	5640000
74	149	150.061	[M-H]-	C9H10O2	p-Coumaryl alcohol	Phenolic acids	302000	334000	302000	495000	466000	486000	25600	36800	28400
75	326.31	325.272	[M+H]+	C20H39NO2	N-Oleoylethanolamine	Others	6030	5950	5820	12800	13100	12400	747	865	697
76	209.2	208.029	[M+H]+	C10H8O5	Fraxetin	Lignans and Coumarins	1460000	1170000	1310000	1430000	1400000	1140000	1710000	1330000	1680000
77	339.1	340.065	[M-H]-	C15H16O9	Esculin(6,7-Dihydroxy Coumarin-6-glucoside)	Lignans and Coumarins	39100	41200	42700	69100	65000	67300	5970	11300	13600
78	741.22	740.181	[M+H]+	C33H40O19	Robinin(Kaempferol-3-O-gal-rham-7-O-rham)	Flavonoids	24900	38200	33200	9	9	9	161000	72100	22600
79	595.2	594.132	[M+H]+	C27H30O15	Apigenin 6,8-C-diglucoside	Flavonoids	105000	129000	116000	119000	100000	103000	118000	100000	101000
80	799.2	800.233	[M-H]-	C36H48O20	Cistanoside A	Phenolic acids	638000	606000	633000	454000	478000	555000	560000	671000	752000
81	665.2	666.184	[M-H]-	C31H38O16	2'-Acetylacteoside	Phenolic acids	1560000	1860000	1810000	106000	47100	101000	4500000	2440000	4070000
82	179.1	178.055	[M+H]+	C10H10O3	Methyl p-coumarate	Phenolic acids	856000	841000	842000	1180000	1110000	1140000	426000	464000	561000
83	177	178.055	[M-H]-	C10H10O3	Trans-4-Hydroxycinnamic Acid Methyl Ester	Phenolic acids	1260000	1320000	1300000	1950000	1950000	1910000	536000	576000	735000
84	209.1	208.064	[M+H]+	C11H12O4	Methyl ferulate	Phenolic acids	2620000	2610000	2570000	1220000	1290000	1210000	5380000	4410000	6370000
85	162.07	161.059	[M+H]+	C6H11NO4	DL-2-Aminoadipic acid	Alkaloids	69700	63800	72800	94000	85800	87500	62900	58100	75800
86	181.06	182.049	[M-H]-	C9H10O4	Syringic Aldehyde	Phenolic acids	379000	319000	365000	549000	499000	529000	140000	128000	126000
87	193.06	194.049	[M-H]-	C10H10O	Trans-ferulic acid	Phenolic	1150000	1150000	1170000	1580000	1510000	1890000	666000	603000	730000

				4		acids									
88	160	161.042	[M-H]-	C9H7NO2	Indole-3-carboxylic acid	Alkaloids	67500	56800	69600	80800	69300	77400	40300	48200	39300
89	314.1	313.117	[M+H]+	C18H19NO4	N-Feruloyltyramine	Alkaloids	11300	11300	10500	16400	20400	17600	4470	5570	6980
90	639.2	640.169	[M-H]-	C29H36O16	Plantamajoside	Phenolic acids	741000	955000	902000	902000	876000	827000	769000	881000	1300000
91	373.11	374.102	[M-H]-	C16H22O10	Geniposidic acid	Terpenoids	12000000	12200000	10300000	167000	263000	202000	20700000	22700000	20800000
92	549.18	550.16	[M-H]-	C23H34O15	Genipin 1-gentiobioside	Terpenoids	40600	42400	49300	49400	75000	76800	38700	35400	40300
93	345.2	346.108	[M-H]-	C15H22O9	Aucubin	Terpenoids	3950000	3940000	3670000	54300	812000	865000	5170000	6120000	5990000
94	255.07	254.049	[M+H]+	C15H10O4	Chrysophanic acid	Quinones	192000	210000	212000	203000	210000	211000	131000	141000	141000
95	609.18	608.146	[M+H]+	C28H32O15	Diosmin	Flavonoids	1440000	1690000	1590000	3060000	2900000	3190000	33500	132000	28900
96	193.07	194.067	[M-H]-	C7H14O6	D-Pinitol	Others	13400	11900	10700	16700	19200	17400	6430	7690	7650
97	179.04	180.035	[M-H]-	C9H8O4	Caffeic acid	Phenolic acids	192000	134000	146000	202000	245000	215000	148000	210000	163000
98	299.12	300.105	[M-H]-	C14H20O7	Salidroside	Phenolic acids	41000	32000	35200	31200	30200	26300	37800	50500	29400
99	137.07	138.061	[M-H]-	C8H10O2	Tyrosol	Phenolic acids	23400	27600	26600	29300	28300	32800	20000	28400	24800
100	303.05	303.04	[M]+	C15H11O7	Delphinidin chloride	Flavonoids	13500	19200	18900	21600	25800	13400	24300	25500	22700
101	469	470.34	[M-H]-	C30H46O4	Hypoglycyrrhizic acid(β)	Terpenoids	20600	20100	19800	9130	9230	11400	41500	55300	35100
102	353.2	352.116	[M+H]+	C21H20O5	1-Methoxyphaseollin	Others	57800	44900	49900	41900	39300	33500	30100	33000	39500
103	479.15	478.125	[M+H]+	C23H26O11	Calceorioside B	Phenolic acids	306000	209000	346000	88600	74300	79600	827000	866000	654000

104	222.09	221.077	[M+H]+	C8H15NO6	N-Acetyl-β-D-mannosamine	Others	18200	20300	18000	23600	16200	18700	21900	22300	23500
105	167.07	168.07	[M-H]-	C9H12O3	Homovanillic alcohol	Phenolic acids	16400	15900	10600	11400	18100	16900	9	9	9
106	463.12	463.124	[M]+	C22H23O11+	Peonidin O-hexoside	Flavonoids	16000	29900	23500	52200	40400	45300	9	9	9
107	136.1	135.049	[M+H]+	C5H5N5	Aminopurine	Alkaloids	245000	303000	281000	488000	548000	487000	34700	55500	36000
108	625.2	624.14	[M+H]+	C28H32O16	Luteolin O-feruloylhexoside	Flavonoids	153000	149000	148000	129000	135000	147000	147000	178000	187000
109	339.1	338.067	[M+H]+	C19H14O6	O-Feruloyl 4-hydroxylcoumarin	Lignans and Coumarins	34400000	33900000	32600000	39200000	35800000	38100000	30000000	32200000	26900000
110	104.1	103.091	[M+H]+	C5H13NO	Choline	Alkaloids	118000	137000	120000	178000	204000	181000	98800	110000	113000
111	235.1	234.123	[M+H]+	C13H18N2O2	N-p-Coumaroyl putrescine	Alkaloids	39900	40100	28800	40400	54100	51600	35100	29800	21400
112	584.2	583.268	[M+H]+	C34H37N3O6	N',N'',N'''-p-Coumaroyl-cinnamoyl-caffeoyl spermidine	Alkaloids	20200	20100	21900	23200	28100	33600	16700	19100	11500
113	337.2	336.16	[M+H]+	C16H24N4O4	N-Sinapoyl agmatine	Alkaloids	15700	21100	20800	21100	24100	24500	5540	6590	9750
114	411.1	410.164	[M+H]+	C23H26N2O5	N',N''-p-Coumaroyl-feruloyl putrescine	Alkaloids	11700	9900	12700	6440	5330	5180	28300	26100	23400
115	477.14	477.14	[M]+	C23H25O11+	Rosinidin O-hexoside	Flavonoids	51800	44900	44100	62200	65100	60400	19700	18900	27400
116	509.1	508.1	[M+H]+	C23H24O13	Syringetin 3-O-hexoside	Flavonoids	422000	504000	446000	46500	46700	43000	1220000	1180000	1370000
117	625.1	624.14	[M+H]+	C28H32O16	Chrysoeriol O-hexosyl-O-hexoside	Flavonoids	43200	34500	45900	56200	61400	69700	9	9	9
118	549.1	548.094	[M+H]+	C25H24O14	Chrysoeriol O-malonylhexoside	Flavonoids	118000	121000	144000	199000	210000	192000	21800	16700	20600
119	655.2	654.132	[M+H]+	C32H30O15	C-Hexosyl-luteolin O-sinapic acid	Flavonoids	8940	8600	9040	15100	14300	21500	4500	2300	2410

120	579.1	578.103	[M+H]+	C26H26O 15	Tricin O-malonylhexoside	Flavonoids	69200	58800	74100	111000	116000	108000	9	9	9
121	493.1	492.105	[M+H]+	C23H24O 12	Tricin 7-O-hexoside	Flavonoids	210000	226000	250000	421000	468000	459000	16400	6950	16300
122	118	117.053	[M+H]+	C8H7N	Indole	Alkaloids	61800	57900	62400	49800	59800	46900	74900	83600	81200
123	474.2	473.144	[M+H]+	C20H23N 7O7	10-Formyl-THF	Alkaloids	1880000	2030000	1970000	1310000	1230000	1410000	3060000	3210000	3160000
124	343.1	342.262	[M+H]+	C19H38N 2O3	Cocamidopropyl βine	Alkaloids	49400	56900	60700	42000	48900	35700	58600	78900	78600
125	207.1	208.064	[M-H]-	C11H12O 4	3,4-Dimethoxycinnami c acid	Phenolic acids	1120000	1120000	1130000	554000	580000	555000	1970000	1800000	2590000
126	485.1	486.114	[M-H]-	C21H26O 13	4-hydroxycoumarin di-glucoside	Lignans and Coumarins	16400	8190	10400	14900	16200	11100	14000	11800	15500
127	315.1	316.065	[M-H]-	C13H16O 9	2,5-Dihydroxy benzoic acid O-hexside	Phenolic acids	4160000	3890000	4250000	4990000	4760000	4310000	2480000	3330000	4090000
128	385.1	386.102	[M-H]-	C17H22O 10	1-O-β-D-Glucopyranos yl sinapate	Phenolic acids	35700	51800	33300	9	9	9	128000	107000	156000
129	465.1	466.073	[M-H]-	C24H18O 10	Cyanidin O-syringic acid	Flavonoids	51100	42300	59900	87000	82000	81300	13300	16900	17200
130	547.11	549.124	[M-2H]-	C25H25O 14+	Peonidin 3-O-(6"-malonylglucos ide)	Flavonoids	64600	52700	59800	64700	64200	64700	21200	53100	26400
131	607.1	608.146	[M-H]-	C28H32O 15	Chrysoeriol 7-O-rutinoside	Flavonoids	60700	81100	61300	129000	109000	116000	9	9	9
132	475.1	476.076	[M-H]-	C22H20O 12	Chrysoeriol O-glucuronic acid	Flavonoids	1050000	1010000	1050000	2020000	1620000	2060000	7270	7030	10600
133	521.1	522.08	[M-H]-	C23H22O 14	Tricin O-saccharic acid	Flavonoids	664000	758000	634000	217000	191000	165000	1400000	1530000	1630000
134	505.1	506.085	[M-H]-	C23H22O 13	Tricin O-glucuronic acid	Flavonoids	383000	397000	416000	832000	656000	829000	9	9	9
135	499.2	500.128	[M-H]-	C22H28O 13	3-O-p-coumaroyl quinic acid O-hexoside	Phenolic acids	5610	6780	6010	7540	8880	6780	9	9	9

136	481.1	482.12	[M-H]-	C22H26O12	5-O-p-Coumaroyl shikimic acid O-hexoside	Phenolic acids	12300	30000	19600	29000	23400	29900	9	9	9
137	337.1	338.085	[M-H]-	C16H18O8	3-O-p-Coumaroyl quinic acid	Phenolic acids	46100	46900	47900	59400	57200	43100	7380	8620	10400
138	359.1	360.088	[M-H]-	C15H20O10	Syringic acid O-glucoside	Phenolic acids	391000	424000	492000	660000	732000	670000	101000	83300	99800
139	329.1	330.061	[M-H]-	C17H14O7	Di-O-methylquercetin	Flavonoids	6830000	6990000	6740000	9440000	10300000	9490000	1410000	3890000	1710000
140	285	286.038	[M-H]-	C15H10O6	Luteolin	Flavonoids	2880	3560	4230	5650	3070	3490	9	9	9
141	123.04	122.032	[M+H]+	C7H6O2	Benzoic acid	Phenolic acids	108000	109000	105000	177000	175000	153000	9	9	9
142	165.03	166.021	[M-H]-	C8H6O4	Terephthalic acid	Phenolic acids	2780000	2550000	2890000	2360000	2340000	2510000	2910000	2710000	3240000
143	431.11	432.088	[M-H]-	C21H20O10	Kaempferol 7-O-rhamnoside	Flavonoids	10900	9120	9860	9	9	9	53100	13900	11000
144	165.05	164.041	[M+H]+	C9H8O3	p-Coumaric acid	Phenolic acids	157000	170000	167000	224000	254000	238000	46700	53500	52000
145	147.12	146.106	[M+H]+	C7H16NO2	Acetylcholine	Alkaloids	19200	21500	20000	27900	34200	32900	10300	9320	13400
146	138.07	137.064	[M+H]+	C7H9N2O	3-Carbamyl-1-methylpyridinium(1-Methylnicotinamide)	Others	14200	9560	10900	17300	13400	13000	15300	14700	15600
147	138	137.042	[M+H]+	C7H7NO2	Trigonelline	Alkaloids	4590	5640	4780	6660	5800	8050	5580	5390	4650
148	139.1	138.026	[M+H]+	C7H6O3	Protocatechuic aldehyde	Flavonoids	49100	83200	102000	141000	151000	124000	9	9	9
149	579.2	578.137	[M+H]+	C27H30O14	Kaempferol 3,7-dirhamnoside(Kaempferitrin)	Flavonoids	256000	245000	258000	9	9	9	1420000	570000	303000
150	131	130.1	[M+H]+	C6H14N2O	N-Acetylputrescine	Alkaloids	37000	34000	41300	23000	27400	20200	70700	57700	51300
151	207.1	208.064	[M-H]-	C11H12O	Sinapinaldehyde	Phenolic	699000	764000	724000	661000	677000	665000	721000	765000	954000

				4		acids								
152	463.12	463.124	[M]+	C22H23O 11+	Peonidin 3-O-glucoside	Flavonoids	32400	26400	33000	38300	44600	45700	9	9	9
153	177.06	178.055	[M-H]-	C10H10O 3	Riboprine	Phenolic acids	3200000	3340000	3500000	4330000	4290000	4230000	1670000	1890000	2280000
154	325.08	324.07	[M+H]+	C15H16O 8	Skimmin	Lignans and Coumarins	2370000	2370000	2510000	2000000	2310000	2700000	1670000	2080000	1840000
155	163.1	162.061	[M+H]+	C10H10O 2	4-MethoxycinnaMalde hyde	Phenolic acids	10900	12000	9280	9010	13200	9810	12900	11800	12000
156	625.18	625.177	[M]+	C28H33O 16+	Peonidin 3,5-O-diglucoside chloride	Flavonoids	9230	8180	7710	4010	5840	6260	12700	9200	13900
157	413.12	414.113	[M-H]-	C22H22O 8	1-O-Feruloyl-3-O-p-C oumaroyl glycerol	Phenolic acids	2470	2600	3010	2790	3090	3090	1650	2390	2090
158	445.1	446.068	[M-H]-	C21H18O 11	Rhein-8-O-D-glucopyr anoside	Quinones	1990000	2070000	1860000	3660000	3210000	3170000	390000	738000	742000
159	315.07	316.065	[M-H]-	C13H16O 9	Protocatechuic acid-4-glucoside	Phenolic acids	6110000	5480000	6560000	6590000	6010000	6990000	4030000	4860000	6280000
160	699.25	700.221	[M-H]-	C32H44O 17	Olivin Diglucoside	Lignans and Coumarins	1310000	1310000	1360000	1710000	1930000	1810000	356000	345000	416000
161	549.16	550.177	[M-H]-	C27H34O 12	Eucommin A	Lignans and Coumarins	63800	64400	65900	42500	36100	39400	93000	115000	103000
162	535.18	536.163	[M-H]-	C26H32O 12	1-Hydroxyterpinin monoglucoside	Lignans and Coumarins	187000	166000	185000	313000	256000	234000	72600	77000	76000
163	519.16	520.168	[M-H]-	C26H32O 11	Terpineol monoglucoside	Lignans and Coumarins	356000	391000	369000	203000	198000	217000	592000	629000	506000
164	325.09	326.085	[M-H]-	C15H18O 8	1-O-[(E)-p-Cumaroyl]-β-D-glucopyranose	Phenolic acids	2090000	1870000	1970000	2380000	3100000	2900000	905000	899000	869000

165	341.08	342.079	[M-H]-	C15H18O9	1-O-[(E)-Caffeoyl]-β-D-glucopyranose	Phenolic acids	3880000	4230000	4220000	1340000	1580000	1680000	6290000	7560000	5970000
166	337.09	338.085	[M-H]-	C16H18O8	3-O-(E)-p-Coumaroyl quinic acid	Phenolic acids	23600	15600	19000	47700	49700	40000	8670	7590	11900
167	517.2	518.137	[M-H]-	C22H30O14	Sibiricose A5	Phenolic acids	250000	252000	272000	179000	181000	241000	348000	346000	431000
168	405.1	406.073	[M-H]-	C19H18O10	Lancerin	Phenolic acids	258000	188000	222000	217000	180000	191000	148000	119000	126000
169	187.1	188.041	[M-H]-	C11H8O3	Ayapin	Lignans and Coumarins	102000	107000	100000	111000	131000	119000	93100	96800	82900
170	417.2	418.142	[M-H]-	C22H26O8	(+)-Syringaresinol	Lignans and Coumarins	74800	67600	71400	93800	92800	97800	8250	7110	9570
171	387.14	388.133	[M-H]-	C21H24O7	Medioresinol	Lignans and Coumarins	37500	37400	33800	50300	46000	45500	6250	7490	6550
172	329.1	330.113	[M-H]-	C15H22O8	3,4,5-Trimethoxyphenyl-β-D-Glucopyranoside	Phenolic acids	18600000	17000000	18400000	25100000	22400000	23700000	11600000	10300000	10600000
173	477.1	478.125	[M-H]-	C23H26O11	3,5-Di-O-galloylshikimic acid	Phenolic acids	42200	39100	39900	48400	49800	47900	8000	12400	16500
174	255.23	256.219	[M-H]-	C16H32O2	Hexadecanoic acid	Phenolic acids	39100000	41200000	44600000	41500000	40000000	51400000	39400000	38400000	38000000
175	609.1	610.126	[M-H]-	C27H30O16	Bioquercetin	Flavonoids	8270	8140	9170	9	9	9	3630	6810	8310
176	389.1	390.096	[M-H]-	C16H22O11	Deacetylasperulosidic acid	Terpenoids	6970	7050	6540	8220	6690	11400	3970	4590	4460
177	487.3	488.317	[M-H]-	C30H48O5	Rutundic acid	Terpenoids	44800	46200	44100	50100	44400	51700	22000	40900	22500
178	577.2	578.137	[M-H]-	C27H30O14	Kaempferol-3,7-O-α-L-rhamnoside	Flavonoids	58700	65700	66900	9	9	9	174000	76000	55500
179	331.1	332.059	[M-H]-	C13H16O10	Glucogallin	Phenolic acids	120000	201000	169000	101000	343000	329000	128000	120000	83700

180	415.1	416.128	[M-H]-	C22H24O8	Apigenin-3-O-α-L-rhamnoside	Flavonoids	20200	20100	21400	26100	21000	22900	22700	19900	18700
181	237.1	238.073	[M-H]-	C12H14O5	Methyl sinapate	Phenolic acids	4600	5190	3990	3360	2670	1880	8390	5020	7210
182	547.2	548.146	[M-H]-	C23H32O15	β-D-Furanofructosyl-α-D-(3-mustard acyl)glucoside	Phenolic acids	62400	70100	72100	106000	98600	108000	7960	6170	5450
183	327.1	328.133	[M-H]-	C16H24O7	3-Hydroxy-4-isopropyl benzylalcohol 3-glucoside	Phenolic acids	191000	163000	155000	295000	283000	261000	9	9	9
184	445.08	446.068	[M-H]-	C21H18O11	Apigenin-7-O-β-D-glucuronide	Flavonoids	437000	380000	423000	945000	763000	759000	11600	17600	21100
185	455.35	456.329	[M-H]-	C30H48O3	24,30-Dihydroxy-12(13)-enolupinol	Terpenoids	144000	113000	109000	225000	185000	291000	50500	62000	51100
186	461.08	462.062	[M-H]-	C21H18O12	Tetahydroxy-flavone-7-O-β-D-glucuronide	Flavonoids	132000	158000	194000	347000	340000	341000	9	9	9
187	469.33	470.309	[M-H]-	C30H46O4	2,3-Dihydroxy 5(6),12(13)diene ursolic acid	Terpenoids	6520	6670	6820	4090	4520	3740	9960	11100	7410
188	471.35	472.323	[M-H]-	C30H48O4	3,24-Dihydroxy-17,21-semiacetal-12(13)oleanolic fruit	Terpenoids	7390000	8990000	8720000	4900000	4690000	5020000	9670000	14300000	11400000
189	471.35	472.323	[M-H]-	C30H48O4	2-Hydroxyoleanolic acid	Terpenoids	8810000	10000000	9150000	4660000	5410000	5410000	10700000	16300000	12000000
190	521.13	522.114	[M-H]-	C24H26O13	Rosmarinyl Glucoside	Phenolic acids	810000	1080000	1090000	1740000	1080000	1570000	283000	272000	375000
191	569.2	570.132	[M-H]-	C25H30O15	Oleuropeinic acid	Terpenoids	23100	38300	23900	16600	13700	13200	30100	28500	28800
192	623.2	624.175	[M-H]-	C29H36O15	Verbascoside	Phenolic acids	29400000	29900000	28700000	7180000	6980000	8720000	49500000	47700000	36900000
193	403.1	404.111	[M-H]-	C17H24O11	Oleoside 11-methyl ester	Phenolic acids	890000	955000	1030000	1200000	1140000	1220000	482000	491000	484000
194	301.07	300.053	[M+H]+	C16H12O	Hispidulin	Flavonoids	58100	65300	60000	81700	70200	70500	9	9	9

				6										
195	331.08	330.061	[M+H]+	C17H14O7	Jaceosidin	Flavonoids	7520	8640	7230	9280	9410	10500	9	9	9
196	463.08	462.062	[M+H]+	C21H18O12	Scutellarin	Flavonoids	215000	190000	251000	376000	340000	338000	9	9	9
197	269.1	268.081	[M+H]+	C13H16O6	2-Feruloyl-sn-glycerol	Phenolic acids	62600	89700	101000	102000	120000	115000	48200	43900	54400
198	519.11	518.085	[M+H]+	C24H22O13	Malonyglygenistin	Flavonoids	56300	57600	62900	86000	99800	89400	9	9	6800
199	223.06	222.044	[M+H]+	C11H10O5	Fraxidin	Lignans and Coumarins	17300	18300	17600	24900	21300	19800	11400	16700	18300
200	301.07	300.053	[M+H]+	C16H12O6	Pratensein	Flavonoids	61400	62400	63200	84400	73400	69200	9	9	9
201	463.08	462.079	[M+H]+	C25H18O9	Luteolin-7-O-glucuronide	Flavonoids	184000	216000	181000	370000	314000	294000	9	9	16200
202	463.12	462.096	[M+H]+	C22H22O11	Diosmetin-7-O-galactoside	Flavonoids	140000	147000	144000	265000	246000	243000	13800	18400	18200
203	477.1	476.076	[M+H]+	C22H20O12	Diosmetin-7-O-glucuronide	Flavonoids	2170000	2730000	3040000	5060000	4110000	4940000	23900	31000	35500
204	549.12	548.094	[M+H]+	C25H24O14	Diosmetin-7-O-(6'-O-malonyl)-β-D-glucoside	Flavonoids	8070	11200	8290	13200	15300	12900	9	9	9
205	595.16	594.132	[M+H]+	C27H30O15	Luteolin-7-O-rutinoside	Flavonoids	35100	37400	41100	9	9	9	219000	71400	50700
206	609.18	608.146	[M+H]+	C28H32O15	Diosmetin-7-O-rutin	Flavonoids	44600	48500	49500	63300	57500	66800	9330	60000	11100
207	331.22	330.199	[M+H]+	C21H30O3	Tussilagonone	Terpenoids	14700	17100	16100	27100	25000	20200	6190	7650	8860
208	479.15	478.125	[M+H]+	C23H26O11	Calceolarioside A	Phenolic acids	1650000	1840000	1890000	331000	317000	322000	5020000	4520000	3410000
209	536.19	518.137	[M+NH4]+	C22H30O14	3'-O-D-glucosylgentiopicroside	Terpenoids	8530	12500	15700	15400	16200	18700	9	9	9

210	637.17	636.14	[M+H]+	C29H32O16	Deglucosylgelidoside	Terpenoids	8980	11000	16900	13400	10900	12400	11900	14300	10700
211	331.17	330.113	[M+H]+	C15H22O8	Bartsioside	Others	158000	113000	146000	243000	247000	275000	9	9	9
212	609.18	608.146	[M+H]+	C28H32O15	Neodiosmin	Flavonoids	36300	59800	51100	70100	65900	76300	23100	58900	9
213	595.17	594.132	[M+H]+	C27H30O15	Vitexin-2-O-D-glucopyranoside	Flavonoids	15500	27200	22400	21700	22200	25200	21100	20200	18200
214	153.05	152.041	[M+H]+	C8H8O3	Dihydro-p-coumarat	Phenolic acids	31000	27300	26200	45700	47100	50600	8000	8950	9830
215	314.1	313.117	[M+H]+	C18H19NO4	Methoxy-N-Caffeoyltyramine	Alkaloids	12000	10900	10800	19600	14200	16000	5720	5020	6290
216	639.2	638.189	[M+H]+	C30H38O15	Cistanoside C	Phenolic acids	130000	167000	187000	19700	11700	34800	370000	536000	278000
217	472.21	472.191	[M]+	C22H34NO10+	Sinapine glucoside	Alkaloids	47800	49300	58200	9	9	9	323000	43500	21000
218	478.29	477.253	[M+H]+	C23H44NO7P	3-{(2-Aminoethoxy)(hydroxy)phosphoryl]oxy}-2-12-octadecadienoate	Alkaloids	6660000	6560000	6460000	2310000	2320000	2180000	13400000	13700000	12900000
219	672.42	671.363	[M+H]+	C31H61O14N	3-Hydroxypropyl palmitate glc-glucosamine	Alkaloids	3860000	3920000	3990000	4390000	4130000	4070000	2250000	2700000	2300000
220	454.3	453.253	[M+H]+	C21H44NO7P	3-{(2-Aminoethoxy)(hydroxy)phosphoryl]oxy}-2-hydroxypropyl palmitate	Alkaloids	485000	391000	368000	199000	212000	212000	709000	1160000	604000
221	518.32	517.276	[M+H]+	C21H48N3O9P	Bis(N,N-diethylethanaminium)-2-acetamido-1,5-anhydro-2-deoxy-1-[-hydroxy(phosphonato)methyl]-D-glucitol	Alkaloids	207000	233000	219000	213000	221000	185000	202000	276000	236000
222	544.34	543.296	[M+H]+	C28H50N	Propyl2-(trimethylam	Others	57800	67300	69400	65400	77500	62800	58000	77200	66700

				O7P	monio)ethyl phosphate									
223	120.08	119.067	[M+H]+	C8H9N	N-Benzylmethylene isomethylamine	Alkaloids	3020000	3320000	3180000	4030000	3710000	3980000	3250000	2600000	3070000
224	653.1	652.1	[M+H]+	C28H28O 18	Chrysoeriol-7-O-[β-D-glucuronopyranosyl-(1→2)-O-β-D-glucurono pyranoside]	Flavonoids	469000	442000	536000	805000	653000	545000	9	9	9
225	623.1	622.091	[M+H]+	C27H26O 17	Apigenin-7-O-[β-D-glucuronopyranosyl(1→2) -O-β-D-glucuronopyra noside)	Flavonoids	748000	701000	806000	1130000	963000	1050000	9	9	9
226	627.2	626.12	[M+H]+	C27H30O 17	6-Hydroxykaempferol-3,6-O-Diglucoside	Flavonoids	21300	22200	30000	24200	19900	17700	30500	24800	18500
227	361	362	[M-H]-	C15H22O 10	Catalpol	Others	405000	590000	555000	542000	710000	604000	505000	736000	588000
228	685	686	[M-H]-	C27H42O 20	Rehmannioside D	Terpenoids	9120000	7830000	8680000	4510000	4010000	3820000	9900000	9350000	8990000

Table. S2. KEGG annotation of 228 secondary metabolites

No.	Compounds	Class	cpd_ID	kegg_map
1	Isorhamnetin-3-O-rutinoside	Flavonoids	--	--
2	3-O-Galloyl-β-D-glucose	Tannins	--	--
3	2-O-Galloyl-β-D-glucose	Tannins	--	--
4	Gallacetophenone	Others	--	--
5	6'-O-glucosylaucubin	Others	--	--
6	Cistanoside F	Phenolic acids	--	--
7	Limocitrin-O-rhamnoside-O-rhamnoside	Flavonoids	--	--
8	Crenatine	Alkaloids	--	--
9	Verbasoside	Phenolic acids	--	--
10	β-Oxoacteoside	Others	--	--
11	Cistanoside D	Others	--	--

12	Isoacteoside	Others	--	--
13	4-Hydroxy-3-methoxycinnamaldehyde	Phenolic acids	--	--
14	Madasiatic acid	Terpenoids	--	--
15	Corosolic acid	Terpenoids	--	--
16	Roseoside	Flavonoids	--	--
17	Phytolaccagenin	Terpenoids	--	--
18	5-(2-Hydroxyethyl)-2-O-glucosylohenol	Phenolic acids	--	--
19	4,5,6-Trihydroxy-2-cyclohexen-1-ylidenea cetonitrile	Alkaloids	--	--
20	Ehretioside	Alkaloids	--	--
21	Tababiphenyl C	Others	--	--
22	Ditartaroyl-hydroxylcoumarin	Phenolic acids	--	--
23	Isorhamnetin acetyl hexoside	Flavonoids	--	--
24	Maleoyl-caffeoylquinic acid	Phenolic acids	--	--
25	Ursolic acid-OCH3	Terpenoids	--	--
26	Feruloylmalic acid	Phenolic acids	--	--
27	Aracarpene 1	Flavonoids	--	--
28	3-O-(2-O-Acetyl-β-D-glucopyranosyl) oleanolic acid	Terpenoids	--	--
29	Methylquercetin rha	Flavonoids	--	--
30	Syringaresinol-4′-O-β-D-monO-glucoside	Lignans and Coumarins	--	--
31	Kaempferol 3-glucuronide-7-glucoside	Flavonoids	--	--
32	Apigenin-7-O-diglucuronide	Flavonoids	--	--
33	Tricin	Flavonoids	C10193	--
34	Kaempferol glc-rha	Flavonoids	--	--
35	(+)-Medioresinol-aceGlu	Lignans and Coumarins	--	--
36	Pinoresinol-Hexose	Lignans and Coumarins	--	--
37	Pinoresinol-acetylglucose	Lignans and Coumarins	--	--
38	Syringaresinol-Hex	Lignans and Coumarins	--	--
39	Syringaresinol-aceGlu	Lignans and Coumarins	--	--
40	Rehmapicroside	Others	--	--
41	Rehmaglutoside D	Terpenoids	--	--

42	Anabasine-Hexose	Alkaloids	--	--
43	Octadecenoic amide	Others	--	--
44	p-Coumaric acid-O-glycoside	Phenolic acids	--	--
45	Feruloyl glucose	Phenolic acids	C17759	--
46	Isosinapic acid-hexoside	Phenolic acids	--	--
47	6,7,8-Tetrahydroxy-5-methoxyflavone	Flavonoids	--	--
48	Coniferaldehyde	Phenolic acids	C02666	ko00940,ko01061,ko01100,ko01110
49	Ferulic acid	Phenolic acids	C01494	ko00940,ko01061,ko01100,ko01110
50	Spermine	Alkaloids	C00750	ko00330,ko00410,ko00480,ko01100,ko04976
51	Syringic acid	Phenolic acids	C10833	ko00627,ko01120
52	Vanillic acid	Phenolic acids	C06672	ko00627,ko01120
53	Baicalin	Flavonoids	--	--
54	Diosmetin	Flavonoids	--	--
55	Coniferyl alcohol	Phenolic acids	C00590	ko00940,ko00998,ko01061,ko01100,ko01110
56	(+)-Piperitol	Lignans and Coumarins	--	--
57	Pinoresinol	Lignans and Coumarins	--	--
58	Indole-5-carboxylic acid	Alkaloids	--	--
59	Indole-3-carboxaldehyde	Alkaloids	C08493	--
60	Pelargonin chloride	Flavonoids	C08725	ko00942
61	2,5-Dihydroxybenzoic acid	Phenolic acids	C00628	ko00350,ko00362,ko00626,ko01100,ko01120
62	Succinic acid	Others	C00042	ko00020,ko00190,ko00250,ko00310,ko00350,ko00360,ko00361,ko00620,ko00630,ko00640,ko00650,ko00720,ko00760,ko00920,ko01060,ko01061,ko01062,ko01063,ko01064,ko01065,ko01066,ko01070,ko01100,ko01110,ko01120,ko01130,ko01200,ko01220,ko02020,ko04024,ko04727,ko04922,ko05230
63	L-(-)-Malic acid	Others	C00497	ko00650,ko01100
64	Citric Acid	Others	C00158	ko00020,ko00250,ko00630,ko00720,ko01060,ko01061,ko01062,ko01063,ko01064,ko01065,ko01066,ko01070,ko01100,ko01110,ko01120,ko01130,ko01200,ko01210,ko01230,ko02020,ko04742,ko04922,ko05230
65	D-Xylonic acid	Others	--	--
66	Vanillin	Phenolic acids	C00755	ko00627,ko00999,ko01061,ko01063,ko01100,ko01110,ko0112

				0
67	Phenethylamine	Others	C05332	ko00360,ko01100
68	4-Hydroxybenzaldehyde	Phenolic acids	C00633	ko00363,ko00623,ko00627,ko01061,ko01100,ko01120
69	N-Acetyl-5-hydroxytryptamine	Alkaloids	C00978	ko00380,ko01100
70	O-Phosphorylethanolamine	Others	--	--
71	Dihydromyricetin(Ampelopsin)	Flavonoids	C02906	ko00941,ko01110
72	4-Hydroxybenzoic acid	Phenolic acids	C00156	ko00130,ko00362,ko00363,ko00623,ko00627,ko00790,ko01061,ko01100,ko01110,ko01120,ko01220,ko07110
73	Coniferin	Phenolic acids	C00761	ko00940
74	p-Coumaryl alcohol	Phenolic acids	C02646	ko00940,ko01061,ko01100,ko01110
75	N-Oleoylethanolamine	Others	--	--
76	Fraxetin	Lignans and Coumarins	C09265	--
77	Esculin(6,7-DihydroxyCoumarin-6-glucoside)	Lignans and Coumarins	C09264	--
78	Robinin(Kaempferol-3-O-gal-rham-7-O-rham)	Flavonoids	C10178	--
79	Apigenin 6,8-C-diglucoside	Flavonoids	--	--
80	Cistanoside A	Phenolic acids	--	--
81	2'-Acetylacteoside	Phenolic acids	--	--
82	Methyl p-coumarate	Phenolic acids	--	--
83	Trans-4-Hydroxycinnamic Acid Methyl Ester	Phenolic acids	--	--
84	Methyl ferulate	Phenolic acids	--	--
85	DL-2-Aminoadipic acid	Alkaloids	--	--
86	Syringic Aldehyde	Phenolic acids	--	--
87	Trans-ferulic acid	Phenolic acids	--	--
88	Indole-3-carboxylic acid	Alkaloids	--	--
89	N-Feruloyltyramine	Alkaloids	--	--
90	Plantamajoside	Phenolic acids	C10485	--
91	Geniposidic acid	Terpenoids	--	--
92	Genipin 1-gentiobioside	Terpenoids	C16965	--
93	Aucubin	Terpenoids	C09771	--

94	Chrysophanic acid	Quinones	--	--
95	Diosmin	Flavonoids	C10039	--
96	D-Pinitol	Others	--	--
97	Caffeic acid	Phenolic acids	C01197	ko00940,ko01061,ko01100,ko01110,ko01220
98	Salidroside	Phenolic acids	C06046	ko00350,ko01100
99	Tyrosol	Phenolic acids	--	--
100	Delphinidin chloride	Flavonoids	--	--
101	Hypoglycyrrhizic acid(β)	Terpenoids	--	--
102	1-Methoxyphaseollin	Others	--	--
103	Calceorioside B	Phenolic acids	--	--
104	N-Acetyl-β-D-mannosamine	Others	--	--
105	Homovanillic alcohol	Phenolic acids	--	--
106	Peonidin O-hexoside	Flavonoids	--	--
107	Aminopurine	Alkaloids	--	--
108	Luteolin O-feruloylhexoside	Flavonoids	--	--
109	O-Feruloyl 4-hydroxylcoumarin	Lignans and Coumarins	--	--
110	Choline	Alkaloids	C00114	ko00260,ko00564,ko01100,ko02010,ko04725,ko04976,ko05231
111	N-p-Coumaroyl putrescine	Alkaloids	C18326	ko00330,ko01100
112	N',N'',N'''-p-Coumaroyl-cinnamoyl-caffeoyl spermidine	Alkaloids	--	--
113	N-Sinapoyl agmatine	Alkaloids	--	--
114	N',N''-p-Coumaroyl-feruloyl putrescine	Alkaloids	--	--
115	Rosinidin O-hexoside	Flavonoids	--	--
116	Syringetin 3-O-hexoside	Flavonoids	--	--
117	Chrysoeriol O-hexosyl-O-hexoside	Flavonoids	--	--
118	Chrysoeriol O-malonylhexoside	Flavonoids	--	--
119	C-Hexosyl-luteolin O-sinapic acid	Flavonoids	--	--
120	Tricin O-malonylhexoside	Flavonoids	--	--
121	Tricin 7-O-hexoside	Flavonoids	--	--
122	Indole	Alkaloids	C00463	ko00380,ko00400,ko00402,ko01100,ko01110,ko04974
123	10-Formyl-THF	Alkaloids	C00234	ko00670,ko00720,ko00970,ko01100,ko01120,ko01200,ko0152

				3
124	Cocamidopropyl βine	Alkaloids	--	--
125	3,4-Dimethoxycinnamic acid	Phenolic acids	--	--
126	4-hydroxycoumarin di-glucoside	Lignans and Coumarins	--	--
127	2,5-Dihydroxy benzoic acid O-hexside	Phenolic acids	--	--
128	1-O-β-D-Glucopyranosyl sinapate	Phenolic acids	--	--
129	Cyanidin O-syringic acid	Flavonoids	--	--
130	Peonidin 3-O-(6"-malonylglucoside)	Flavonoids	--	--
131	Chrysoeriol 7-O-rutinoside	Flavonoids	--	--
132	Chrysoeriol O-glucuronic acid	Flavonoids	--	--
133	Tricin O-saccharic acid	Flavonoids	--	--
134	Tricin O-glucuronic acid	Flavonoids	--	--
135	3-O-p-coumaroyl quinic acid O-hexoside	Phenolic acids	--	--
136	5-O-p-Coumaroyl shikimic acid O-hexoside	Phenolic acids	--	--
137	3-O-p-Coumaroyl quinic acid	Phenolic acids	C12208	ko00940,ko00941,ko00945,ko01110
138	Syringic acid O-glucoside	Phenolic acids	--	--
139	Di-O-methylquercetin	Flavonoids	C01265	ko00944
140	Luteolin	Flavonoids	C01514	ko00941,ko00944,ko01100,ko01110
141	Benzoic acid	Phenolic acids	C00180	ko00360,ko00362,ko00621,ko00623,ko00627,ko01063,ko01100,ko01110,ko01120,ko01220
142	Terephthalic acid	Phenolic acids	C06337	ko00624,ko00627,ko01100,ko01120,ko01220
143	Kaempferol 7-O-rhamnoside	Flavonoids	--	--
144	p-Coumaric acid	Phenolic acids	C00811	ko00130,ko00350,ko00940,ko00950,ko00999,ko01061,ko01100,ko01110,ko01220
145	Acetylcholine	Alkaloids	C08201	--
146	3-Carbamyl-1-methylpyridinium(1-Methyl nicotinamide)	Others	C02918	ko00760,ko01100,ko04976
147	Trigonelline	Alkaloids	C01004	ko00760
148	Protocatechuic aldehyde	Flavonoids	C16700	ko00950,ko01063
149	Kaempferol 3,7-dirhamnoside(Kaempferitrin)	Flavonoids	C16981	--

150	N-Acetylputrescine	Alkaloids	C02714	ko00330,ko01100
151	Sinapinaldehyde	Phenolic acids	--	--
152	Peonidin 3-O-glucoside	Flavonoids	C12141	ko00942
153	Riboprine	Phenolic acids	--	--
154	Skimmin	Lignans and Coumarins	--	--
155	4-MethoxycinnaMaldehyde	Phenolic acids	--	--
156	Peonidin 3,5-O-diglucoside chloride	Flavonoids	--	--
157	1-O-Feruloyl-3-O-p-Coumaroyl glycerol	Phenolic acids	--	--
158	Rhein-8-O-D-glucopyranoside	Quinones	--	--
159	Protocatechuic acid-4-glucoside	Phenolic acids	--	--
160	Olivin Diglucoside	Lignans and Coumarins	--	--
161	Eucommin A	Lignans and Coumarins	C10560	--
162	1-Hydroxyterpinin monoglucoside	Lignans and Coumarins	--	--
163	Terpineol monoglucoside	Lignans and Coumarins	--	--
164	1-O-[(E)-p-Cumaroyl]-β-D-glucopyranose	Phenolic acids	--	--
165	1-O-[(E)-Caffeoyl]-β-D-glucopyranose	Phenolic acids	--	--
166	3-O-(E)-p-Coumaroyl quinic acid	Phenolic acids	--	--
167	Sibiricose A5	Phenolic acids	--	--
168	Lancerin	Phenolic acids	C10075	--
169	Ayapin	Lignans and Coumarins	C18078	--
170	(+)-Syringaresinol	Lignans and Coumarins	C10889	--
171	Medioresinol	Lignans and Coumarins	--	--
172	3,4,5-Trimethoxyphenyl-β-D-Glucopyranoside	Phenolic acids	--	--
173	3,5-Di-O-galloylshikimic acid	Phenolic acids	--	--
174	Hexadecanoic acid	Phenolic acids	C00249	ko00061,ko00062,ko00071,ko00073,ko01040,ko01060,ko01100,ko01212
175	Bioquercetin	Flavonoids	--	--
176	Deacetylasperulosidic acid	Terpenoids	--	--
177	Rutundic acid	Terpenoids	--	--
178	Kaempferol-3,7-O-α-L-rhamnoside	Flavonoids	--	--
179	Glucogallin	Phenolic acids	--	--

180	Apigenin-3-O-α-L-rhamnoside	Flavonoids	--	--
181	Methyl sinapate	Phenolic acids	--	--
182	β-D-Furanofructosyl-α-D-(3-mustard acyl)glucoside	Phenolic acids	--	--
183	3-Hydroxy-4-isopropylbenzylalcohol 3-glucoside	Phenolic acids	--	--
184	Apigenin-7-O-β-D-glucuronide	Flavonoids	--	--
185	24,30-Dihydroxy-12(13)-enolupinol	Terpenoids	--	--
186	Tetahydroxy-flavone-7-O-β-D-glucuronide	Flavonoids	--	--
187	2,3-Dihydroxy 5(6),12(13)diene ursolic acid	Terpenoids	--	--
188	3,24-Dihydroxy-17,21-semiacetal-12(13)oleanolic fruit	Terpenoids	--	--
189	2-Hydroxyoleanolic acid	Terpenoids	--	--
190	Rosmarinyl Glucoside	Phenolic acids	--	--
191	Oleuropeinic acid	Terpenoids	--	--
192	Verbascoside	Phenolic acids	C10501	--
193	Oleoside 11-methyl ester	Phenolic acids	--	--
194	Hispidulin	Flavonoids	C10058	--
195	Jaceosidin	Flavonoids	--	--
196	Scutellarin	Flavonoids	--	--
197	2-Feruloyl-sn-glycerol	Phenolic acids	--	--
198	Malonyglygenistin	Flavonoids	--	--
199	Fraxidin	Lignans and Coumarins	C17479	--
200	Pratensein	Flavonoids	C10520	ko00943
201	Luteolin-7-O-glucuronide	Flavonoids	--	--
202	Diosmetin-7-O-galactoside	Flavonoids	--	--
203	Diosmetin-7-O-glucuronide	Flavonoids	--	--
204	Diosmetin-7-O-(6'-O-malonyl)-β-D-glucoside	Flavonoids	--	--
205	Luteolin-7-O-rutinoside	Flavonoids	--	--
206	Diosmetin-7-O-rutin	Flavonoids	--	--

207	Tussilagonone	Terpenoids	--	--
208	Calceolarioside A	Phenolic acids	--	--
209	3'-O-D-glucosylgentiopicroside	Terpenoids	--	--
210	Deglucosylgelidoside	Terpenoids	--	--
211	Bartsioside	Others	--	--
212	Neodiosmin	Flavonoids	--	--
213	Vitexin-2-O-D-glucopyranoside	Flavonoids	--	--
214	Dihydro-p-coumarat	Phenolic acids	--	--
215	Methoxy-N-Caffeoyltyramine	Alkaloids	--	--
216	Cistanoside C	Phenolic acids	--	--
217	Sinapine glucoside	Alkaloids	--	--
218	3-{(2-Aminoethoxy)(hydroxy)phosphoryl] oxy}-2-12-octadecadienoate	Alkaloids	--	--
219	3-Hydroxypropyl palmitate glc-glucosamine	Alkaloids	--	--
220	3-{(2-Aminoethoxy)(hydroxy)phosphoryl] oxy}-2-hydroxypropyl palmitate	Alkaloids	--	--
221	Bis(N,N-diethylethanaminium)-2-acetamido-1,5-anhydro-2-deoxy-1-[-hydroxy(phosphonato)methyl]-D-glucitol	Alkaloids	--	--
222	Propyl2-(trimethylammonio)ethyl phosphate	Others	--	--
223	N-Benzylmethylene isomethylamine	Alkaloids	--	--
224	Chrysoeriol-7-O-[β-D-glucuronopyranosyl-(1→2)-O-β-D-glucuronopyranoside]	Flavonoids	--	--
225	Apigenin-7-O-[β-D-glucuronopyranosyl(1→2)-O-β-D-glucuronopyranoside)	Flavonoids	--	--
226	6-Hydroxykaempferol-3,6-O-Diglucoside	Flavonoids	--	--
227	Catalpol	Others	--	--
228	Rehmannioside D	Terpenoids	--	--

Table. S3. KEGG annotation of 58 differential metabolites

NO.	Compounds	Class	VIP	Fold_Change	Type	kegg_map
1	Limocitrin-O-rhamnoside-O-rhamnoside	Flavonoids	1.33	102.48	up	--
2	Phytolaccagenin	Terpenoids	1.93	0.00	down	--
3	Ehretioside	Alkaloids	1.90	12259.26	up	--
4	Aracarpene 1	Flavonoids	1.95	21777.78	up	--
5	Kaempferol 3-glucuronide-7-glucoside	Flavonoids	1.86	8529.63	up	--
6	Apigenin-7-O-diglucuronide	Flavonoids	2.07	79666.67	up	--
7	Tricin	Flavonoids	1.68	1644.44	up	--
8	Kaempferol glc-rha	Flavonoids	1.93	16814.81	up	--
9	6,7,8-Tetrahydroxy-5-methoxyflavone	Flavonoids	1.85	8066.67	up	--
10	Baicalin	Flavonoids	1.17	34.65	up	--
11	Diosmetin	Flavonoids	1.95	21444.44	up	--
12	Pelargonin chloride	Flavonoids	1.80	0.00	down	ko00942
13	p-Coumaryl alcohol	Phenolic acids	1.03	15.94	up	ko00940,ko01061,ko01100,ko01110
14	N-Oleoylethanolamine	Others	1.04	16.59	up	--
15	Robinin(Kaempferol-3-O-gal-rham-7-O-rham)	Flavonoids	1.83	0.00	down	--
16	2'-Acetylacteoside	Phenolic acids	1.19	0.02	down	--
17	Geniposidic acid	Terpenoids	1.33	0.01	down	--
18	Diosmin	Flavonoids	1.23	47.07	up	--
19	Homovanillic alcohol	Phenolic acids	1.68	1718.52	up	--
20	Peonidin O-hexoside	Flavonoids	1.80	5107.41	up	--
21	Syringetin 3-O-hexoside	Flavonoids	1.13	0.04	down	--
22	Chrysoeriol O-hexosyl-O-hexoside	Flavonoids	1.84	6937.04	up	--
23	Tricin O-malonylhexoside	Flavonoids	1.90	12407.41	up	--
24	Tricin 7-O-hexoside	Flavonoids	1.16	34.00	up	--
25	1-O-β-D-Glucopyranosyl sinapate	Phenolic acids	1.91	0.00	down	--
26	Chrysoeriol 7-O-rutinoside	Flavonoids	1.90	13111.11	up	--
27	Chrysoeriol O-glucuronic acid	Flavonoids	1.44	228.92	up	--
28	Tricin O-glucuronic acid	Flavonoids	2.08	85814.81	up	--
29	3-O-p-coumaroyl quinic acid O-hexoside	Phenolic acids	1.60	859.26	up	--

30	5-O-p-Coumaroyl shikimic acid O-hexoside	Phenolic acids	1.75	3048.15	up	--
31	Luteolin	Flavonoids	1.52	452.22	up	ko00941,ko00944,ko01100,ko01110
32	Benzoic acid	Phenolic acids	1.94	18703.70	up	ko00360,ko00362,ko00621,ko00623,ko00627,ko01063,ko01100,ko01110,ko01120,ko01220
33	Kaempferol 7-O-rhamnoside	Flavonoids	1.71	0.00	down	--
34	Protocatechuic aldehyde	Flavonoids	1.92	15407.41	up	ko00950,ko01063
35	Kaempferol 3,7-dirhamnoside(Kaempferitrin)	Flavonoids	2.06	0.00	down	--
36	Peonidin 3-O-glucoside	Flavonoids	1.80	4762.96	up	ko00942
37	Bioquercetin	Flavonoids	1.57	0.00	down	--
38	Kaempferol-3,7-O-α-L-rhamnoside	Flavonoids	1.87	0.00	down	--
39	β-D-Furanofructosyl-α-D-(3-mustard acyl)glucoside	Phenolic acids	1.03	15.97	up	--
40	3-Hydroxy-4-isopropylbenzylalcohol 3-glucoside	Phenolic acids	1.99	31074.07	up	--
41	Apigenin-7-O-β-D-glucuronide	Flavonoids	1.22	49.05	up	--
42	Tetahydroxy-flavone-7-O-β-D-glucuronide	Flavonoids	2.01	38074.07	up	--
43	Hispidulin	Flavonoids	1.85	8237.04	up	--
44	Jaceosidin	Flavonoids	1.63	1081.11	up	--
45	Scutellarin	Flavonoids	2.01	39037.04	up	--
46	Malonyglygenistin	Flavonoids	1.51	40.36	up	--
47	Pratensein	Flavonoids	1.86	8407.41	up	ko00943
48	Luteolin-7-O-glucuronide	Flavonoids	1.61	60.30	up	--
49	Diosmetin-7-O-galactoside	Flavonoids	1.02	14.96	up	--
50	Diosmetin-7-O-glucuronide	Flavonoids	1.39	156.08	up	--
51	Diosmetin-7-O-(6'-O-malonyl)-β-D-glucoside	Flavonoids	1.67	1533.33	up	--
52	Luteolin-7-O-rutinoside	Flavonoids	1.87	0.00	down	--
53	3'-O-D-glucosylgentiopicroside	Terpenoids	1.69	1862.96	up	--

54	Bartsioside	Others	1.98	28333.33	up	--
55	Cistanoside C	Phenolic acids	1.04	0.06	down	--
56	Sinapine glucoside	Alkaloids	1.83	0.00	down	--
57	Chrysoeriol-7-O-[β-D-glucuronopyranosyl-(1→2)-O-β-D-glucuronopyranoside]	Flavonoids	2.07	74185.19	up	--
58	Apigenin-7-O-[β-D-glucuronopyranosyl(1→2)-O-β-D-glucuronopyranoside)	Flavonoids	2.11	116407.41	up	--
